# Supplementary material for: Eomes directs the formation of spatially and functionally diverse extraembryonic hematovascular tissues
Source: Dev Cell. Author manuscript; Available in PMC 2025 Dec 11. (PMC7618462; doi:10.1016/j.devcel.2025.06.001)
Supplement: Supplementary Materials [file EMS211404-supplement-Supplementary_Materials.zip › 1-s2.0-S1534580725003338-mmc1.pdf]

Developmental Cell, Volume *60*

Supplemental information

Eomes directs the formation of spatially and functionally diverse extraembryonic hematovascular tissues

Bart Theeuwes, Luke T.G. Harland, Alexandra M. Bisia, Ita Costello, Mai-Linh N. Ton, Tim Lohoff, Stephen J. Clark, Ricard Argelaguet, Nicola K. Wilson, Wolf Reik, Elizabeth K. Bikoff, Elizabeth J. Robertson, and Berthold Göttgens

Supplementary Figure 1: Multiome quality control, related to Figure 1.

A) Violin plots displaying the natural log(number of counts), natural log(number of genes), percentage of mitochondrial and ribosomal counts per sample. WT and Eomes-KD samples are colored in shades of black and red, respectively.

B) Violin plots displaying natural log(number of fragments) and Transcription Start Sites (TSS) enrichment score, samples colored as in A.

C) Genome wide normalized enrichment score around TSS (top) and Fragment length distribution per sample (bottom), samples colored as in A.

D) Bar graph displaying number of chromatin peaks per genomic feature.

E) Transferred cell type label from integration with scRNA-seq gastrulation atlas[17] for each of the final cell type labels.

F) Projection of in vitro data on the scRNA-seq gastrulation atlas UMAP[17], colored by transferred cell type label. Cell types coloured as in E.

G) *In vitro* UMAP colored by transferred cell type label. Cell types coloured as in E.

H) Expression pattern of marker genes from Fig 1C per cell type in the *in vivo* multiome gastrulation atlas[18].

I) ChromVAR pattern of marker genes from Fig 1D per cell type in the *in vivo* multiome gastrulation atlas[18].

SUPP. FIGURE 2

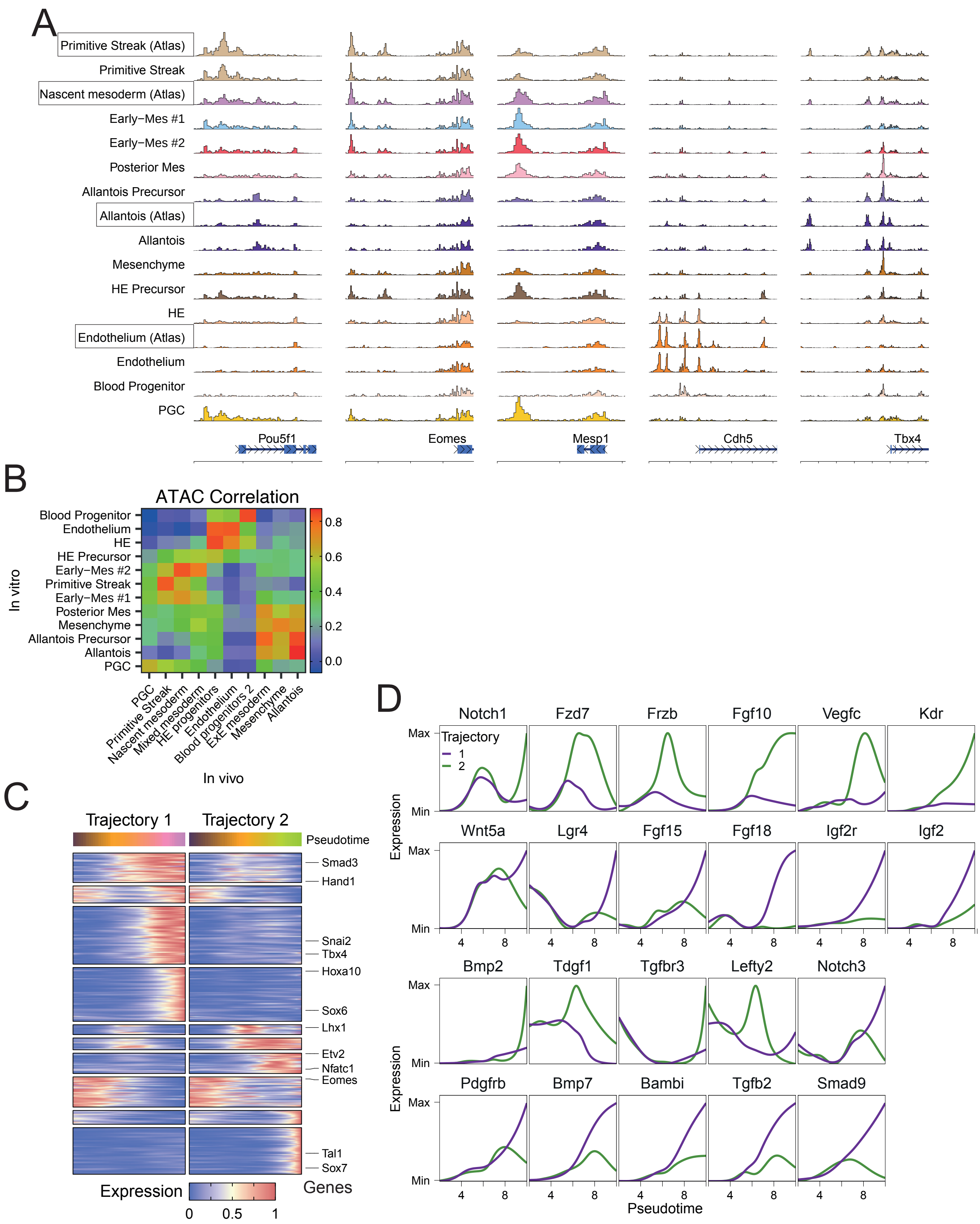

Supplementary Figure 2: EB differentiation additional information, related to Figure 1.

A) Chromatin accessibility around marker genes for PS (Pou5f1), Early Mesoderm (Eomes/Mesp1), Endothelium (Cdh5), and Allantois (Tbx4). EB cell types are compared to a subset of cell types from a mouse gastrulation *in vivo* multiomic dataset (labelled as Atlas)[18].

B) Comparison between accessibility profiles of *in vitro* cell types and *in vivo* cell types from an *in vivo* multiome gastrulation atlas[18] using cell type marker peaks identified for the *in vivo* cell types, colored by pearson correlation score.

C) Heatmap showing normalized gene expression along pseudo-time for both trajectories, clustered by expression patterns. Differential testing was performed on pseudo-time range indicated in Fig 1F.

D) Gene expression (solid lines) for allantois (purple – trajectory 1) and YS (green – trajectory 2) trajectories plotted along pseudo-time.

SUPP. FIGURE 3

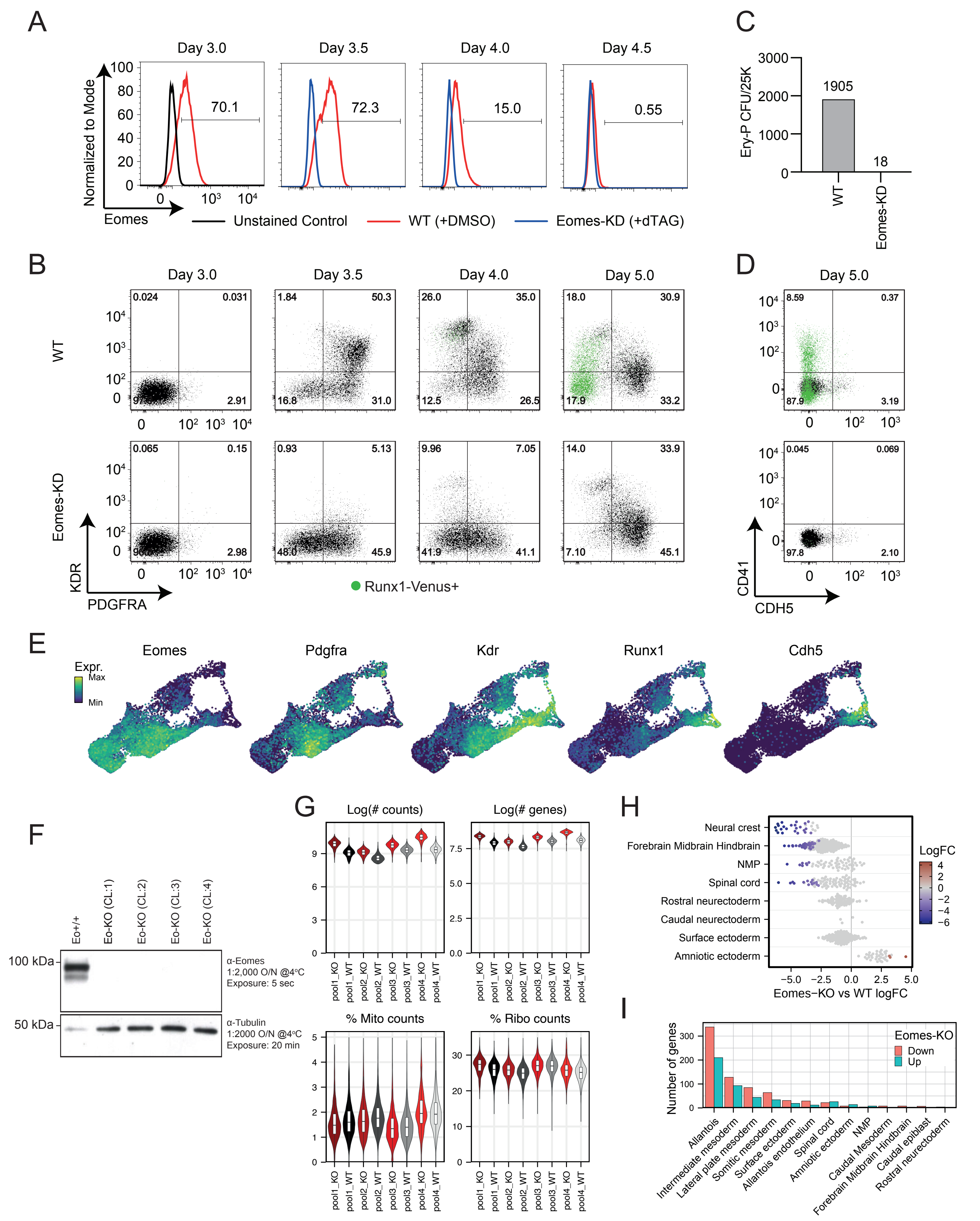

Supplementary Figure 3: Eomes-KD EB differentiation additional information, related to Figure 2 and 3.

A) Intracellular flow cytometric analyses of Eomes protein expression at different timepoints of EB differentiation. The black line at day 3 indicates the signal for the unstained control. EB cultures were treated with DMSO (red lines) and dTAG13 (blue lines) from day 3-4.

B) Flow cytometric analyses of KDR and PDGFRA expression at different timepoints of WT (top) or Eomes-KD (bottom) EB cultures. Green dots indicate Runx1-Venus+ cells.

C) Primitive erythrocyte colony forming units counted after 5 days of growth in Methocult M3434. Cells were plated at day 5 from single cell suspensions of either WT or Eomes-KD EBs. Ery-P; Primitive Erythrocytes, CFU; Colony Forming Units, 25K; 25,000 cells.

D) Flow cytometric analyses of CD41 and CDH5 expression at day 5 of WT (top) or Eomes-KD (bottom) EB culture when DMSO or dTAG13 weas added to cultures from days 3-4. Green dots indicate Runx1-Venus+ cells.

E) UMAPs of WT multiome cultures colored by expression levels of indicated genes.

F) Western blot for Eomes and α-tubulin expression in tdTomato+ Eomes (Eo) +/+ parental and four KO clones collected at day 4 of EB differentiation, confirming Eomes-KO for all four clones used in chimera-seq experiments displayed in Fig 3.

G) Violin plots displaying natural log(number of counts), natural log(number of genes), percentage of mitochondrial and ribosomal counts per sample of Eomes-KO chimera-seq, with shades of black and red indicating WT host Eomes-KO samples, respectively.

H) Differential abundance for Eomes-KO as in Fig 3D, for ectodermal lineages. Negative logFC values indicate depletion of KO cells, while positive logFC values indicate enrichment, neighborhoods without statistical significance are colored grey.

I) Number of detected differentially expressed genes that are up (blue) and down (red) regulated in Eomes-KO compared to WT for each cell type.

SUPP. FIGURE 4

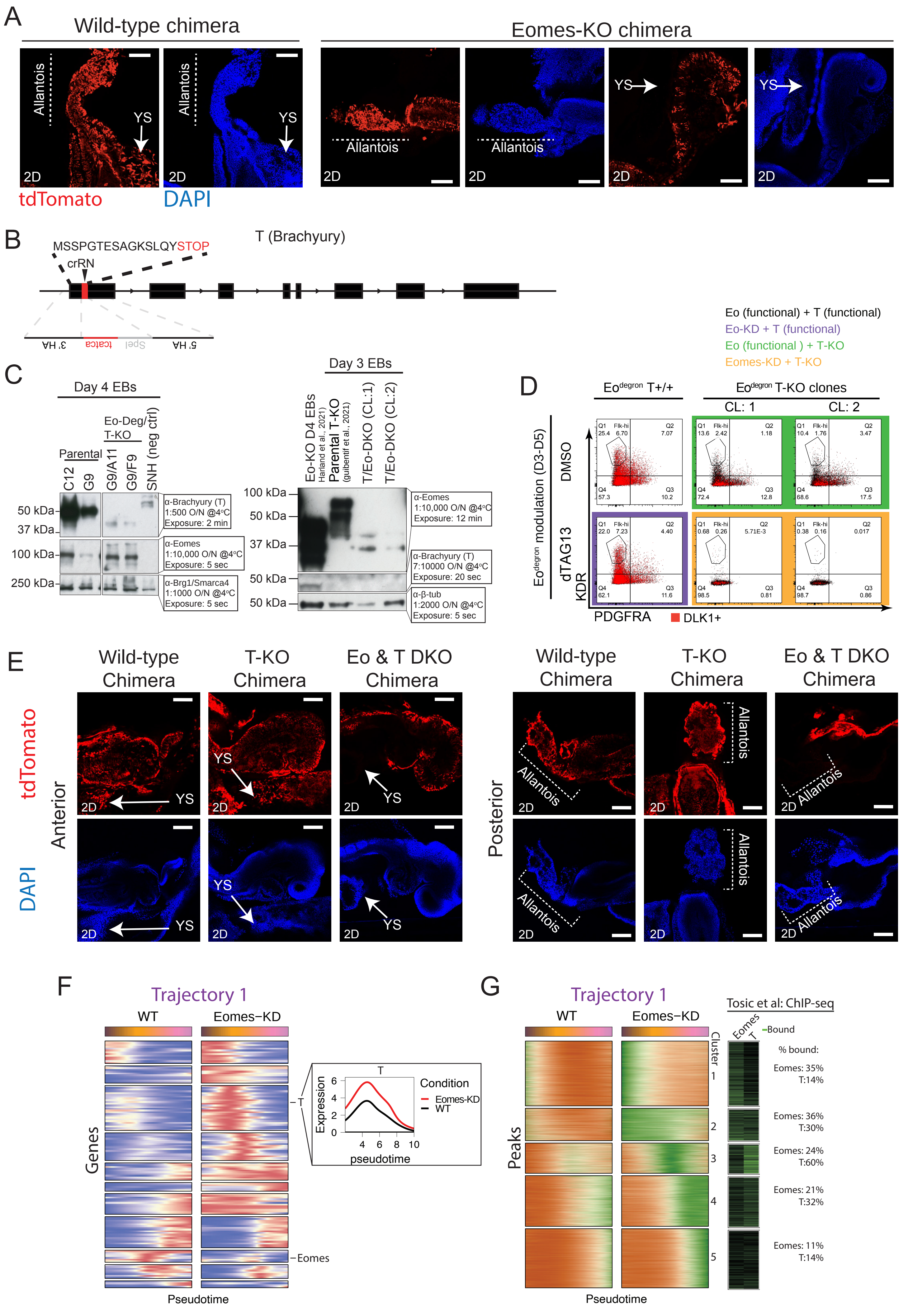

Supplementary Figure 4: Eomes and T Chimera-seq additional information, related to Figure 3 and 4.

A) Single-channel tdTomato and DAPI images of WT and Eomes Chimeric embryos from Fig 3D. Scale bars; 200 μm

B) KO strategy for T-KO used for generation of the Eo-Deg/T-KO ESC lines.

C) Western blot for T, Eomes, and Smarca4 or α-β-tubulin (loading controls) protein expression collected from day 3 and 4 EBs (as indicated) to confirm correct generation of T-KO in Eo-Deg/T-KO (left) and Eomes-KO in Eo/T-dKO lines (right).

D) Flow cytometric analyses of PDGFRA and KDR expression for $Eomes^{WT}/T^{WT}$, $Eomes^{KD}/T^{WT}$, $Eomes^{WT}/T^{KO}$, $Eomes^{KD}/T^{KO}$ at day 5 of EB differentiation. Red dots indicate DLK1 + cells. dTAG or DMSO was added from day 3 onwards.

E) Single-channel tdTomato and DAPI images from anterior (left) and posterior (right) of WT, T, and Eomes/T double KO Chimeric embryos from Fig 4D. Scale bars; 200 μm

F) Differential expression of genes between the Eomes-KD and WT conditions along the allantois trajectory (left), with normalized expression of T highlighted (right).

G) Differential accessibility of peaks between the Eomes-KD and WT conditions along the allantois trajectory (left) alongside Eomes and T ChIP seq binding to those peaks (right) from Tosic et al.[13].
