# Supplementary material for: Eomes directs the formation of spatially and functionally diverse extraembryonic hematovascular tissues
Source: Dev Cell. Author manuscript; Available in PMC 2025 Dec 11. (PMC7618462; doi:10.1016/j.devcel.2025.06.001)
Supplement: Supplementary Materials [file EMS211404-supplement-Supplementary_Materials.zip › 1-s2.0-S1534580725003338-mmc6.pdf]

Short article

Developmental Cell

Eomes directs the formation of spatially and functionally diverse extraembryonic hematovascular tissues

Graphical abstract

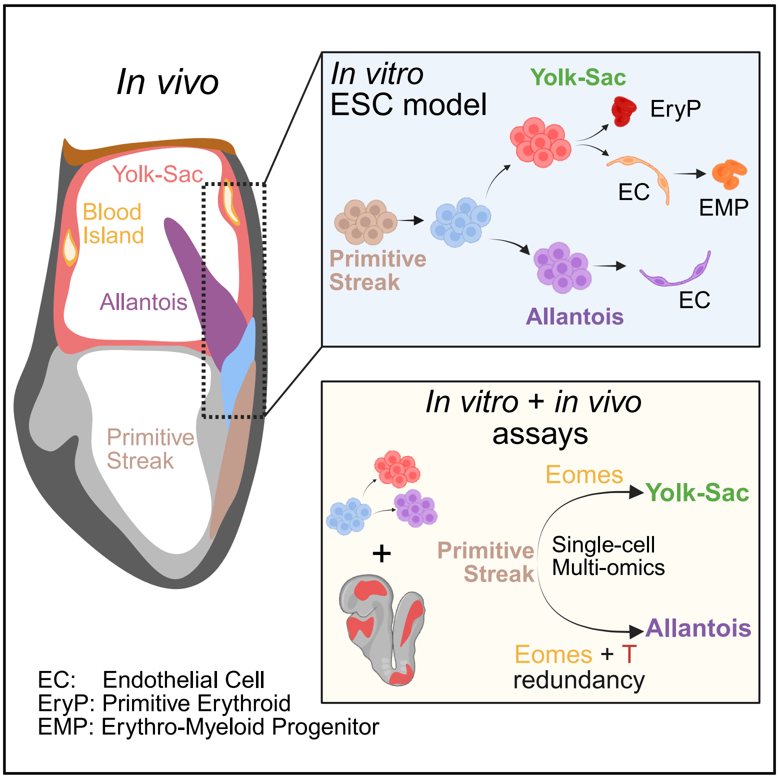

Highlights

- ESC-differentiation system mimics extraembryonic mesoderm development
- Identification of gene networks coordinating yolk sac and allantois formation
- Eomes is essential for the development of early yolk-sac mesoderm progenitors
- Eomes and T double knockout impedes yolk sac and allantois formation

Authors

Bart Theeuwes, Luke T.G. Harland, Alexandra M. Bisia, ..., Elizabeth K. Bikoff, Elizabeth J. Robertson, Berthold Göttgens

Correspondence

ltgh2@cam.ac.uk (L.T.G.H.), elizabeth.robertson@path.ox.ac.uk (E.J.R.), bg200@cam.ac.uk (B.G.)

In brief

Theeuwes, Harland, et al. show that an ESC-derived differentiation system mimics the formation of extraembryonic mesodermal tissues, including the yolk sac and allantois. Using this system alongside *in vivo* models, they identify novel requirements for Eomes and Brachyury during early steps in extraembryonic mesoderm diversification.

Theeuwes et al., 2025, Developmental Cell *60*, 2703–2714
October 20, 2025 © 2025 The Author(s). Published by Elsevier Inc.
https://doi.org/10.1016/j.devcel.2025.06.001

CellPress

Developmental Cell

CellPress
OPEN ACCESS

Short article

Eomes directs the formation of spatially and functionally diverse extraembryonic hematovascular tissues

Bart Theeuwes,[1,2,8] Luke T.G. Harland,[1,2,3,8,*] Alexandra M. Bisia,[4] Ita Costello,[4] Mai-Linh N. Ton,[1,2] Tim Lohoff,[5] Stephen J. Clark,[6,7] Ricard Argelaguet,[5,7] Nicola K. Wilson,[1,2] Wolf Reik,[5,6,7] Elizabeth K. Bikoff,[4] Elizabeth J. Robertson,[4,*] and Berthold Göttgens[1,2,9,*]
[1]Cambridge Stem Cell Institute, University of Cambridge, Cambridge, UK
[2]Department of Haematology, University of Cambridge, Cambridge, UK
[3]Wolfson College, University of Cambridge, Cambridge, UK
[4]Sir William Dunn School of Pathology, University of Oxford, Oxford, UK
[5]Epigenetics Programme, Babraham Institute, Cambridge, UK
[6]Wellcome Trust Sanger Institute, Hinxton, Cambridge, UK
[7]Present address: Altos Labs Cambridge Institute of Science, Granta Park, Cambridge, UK
[8]These authors contributed equally
[9]Lead contact
*Correspondence: ltgh2@cam.ac.uk (L.T.G.H.), elizabeth.robertson@path.ox.ac.uk (E.J.R.), bg200@cam.ac.uk (B.G.)
https://doi.org/10.1016/j.devcel.2025.06.001

SUMMARY

During mouse gastrulation, extraembryonic mesoderm (ExEM) contributes to the extraembryonic yolk sac (YS) and allantois, both of which are essential for successful gestation. Although the genetic networks coordinating intra-embryonic mesodermal subtype specification are well studied, ExEM diversification remains poorly understood. Here, we identify that embryoid body (EB) *in vitro* differentiation generates distinct lineages of mesodermal cells, matching YS and allantois development. Combining *in vitro* and *in vivo* mouse models, we discover that Eomesodermin (Eomes) controls the formation of YS-fated ExEM but is dispensable for allantois formation. Furthermore, simultaneous disruption of Eomes and T impedes the specification of any YS or allantois mesoderm, indicating compensatory roles for T during allantois formation upon Eomes depletion. Our study highlights previously unrecognized functional and mechanistic diversity in ExEM diversification and endothelial development and introduces a tractable EB model to dissect the signaling pathways and transcriptional networks driving the formation of key extraembryonic tissues.

INTRODUCTION

At the onset of murine gastrulation, extraembryonic mesodermal (ExEM) cells originating from the posterior region of the primitive streak (PS) migrate proximally to form hematovascular and mesenchymal cell types in both the visceral yolk sac (YS) and allantois (Figure 1A). These ExEM cells contribute to the formation of the YS blood islands, which contain primitive erythrocytes and endothelial cells by embryonic day (E)7.5.[1] As development progresses, YS endothelial progenitors expand to form an intricate vascular plexus, which plays a dual role in regulating nutrient exchange and serves as a site of pro-definitive hematopoiesis from E8.25.[2]

By approximately E7.5, the allantoic bud begins to emerge from ExEM, traversing the posterior PS. The allantois extends into the exocoelomic cavity and, by E8.5, fuses with the chorionic plate, giving rise to the umbilical cord and fetal placental vasculature.[3–5] Both the allantois and YS are major sites of *de novo* vasculogenesis during murine gastrulation.[4,5] The molecular pathways regulating the initial diversification of ExEM into YS and allantois lineages, however, remain poorly understood.

T-box transcription factors (TFs) Eomesodermin (Eomes) and Brachyury (T) are dynamically expressed in the epiblast, PS, and ExEM precursors at the onset of murine gastrulation.[6–11] Disrupting the functionality of Eomes or T has demonstrated their essential and partially redundant roles during gastrulation.[8–10,12,13] T is dispensable for the initial specification of anterior intraembryonic mesoderm and ExEM but is required for sustained posterior mesoderm development and axial elongation,[14–16] while Eomes is crucial for specifying cardiac mesoderm and the definitive endoderm lineage.[8,9] Using an *in vitro* model of hematovascular formation, we recently demonstrated that Eomes is necessary for YS hematopoiesis and hemogenic endothelial formation but dispensable for non-hemogenic endothelium.[10] While *in vivo* fate mapping experiments demonstrate that Eomes+ cells contribute to the allantois,[10] the role of Eomes during allantois formation has not been investigated. More broadly, the underlying Eomes-dependent mechanisms that coordinate the formation of

This is an open access article under the CC BY license (http://creativecommons.org/licenses/by/4.0/).

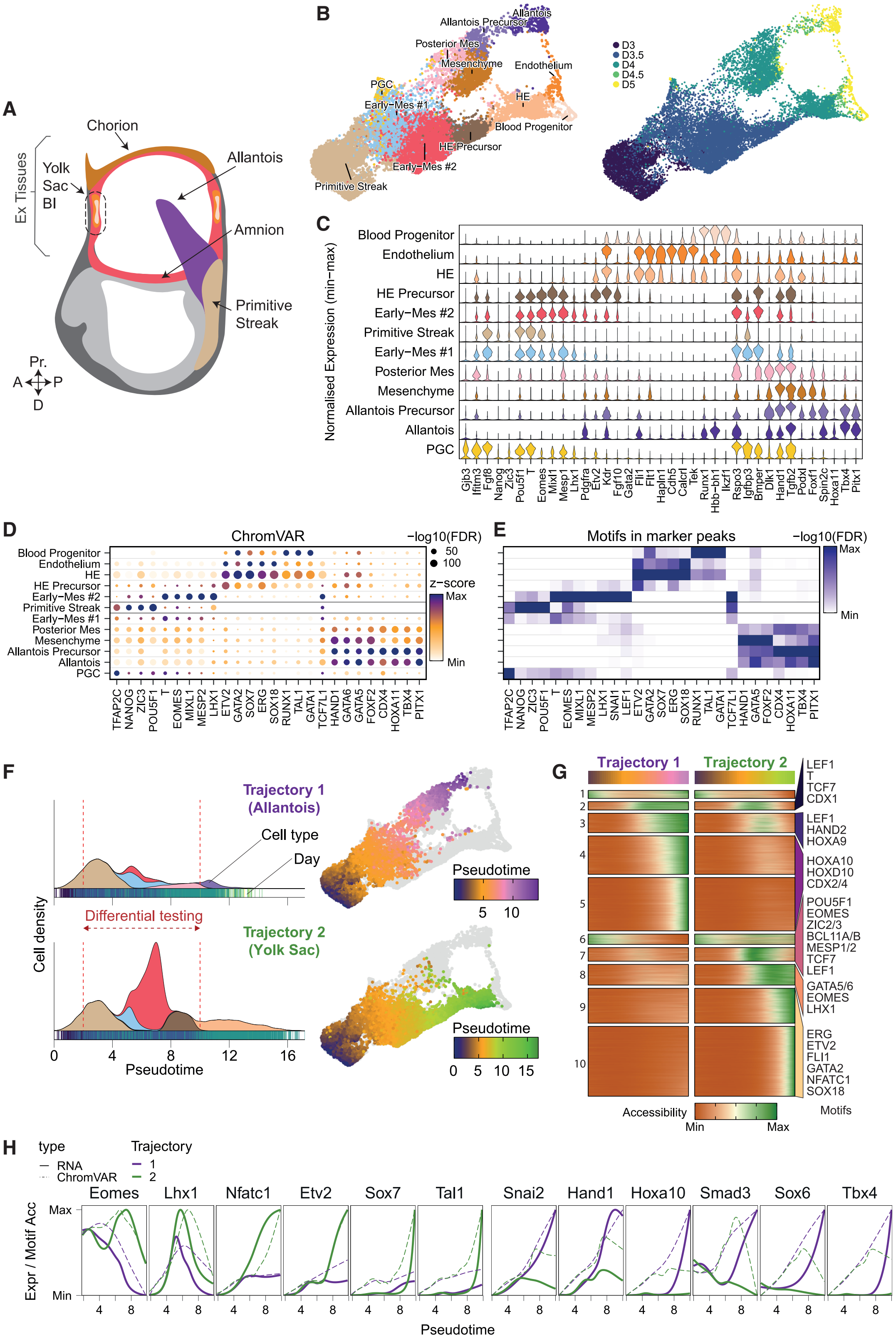

(legend on next page)

CellPress
OPEN ACCESS

early hematovascular lineages remain poorly understood, and a cell-autonomous role for Eomes during blood and endothelial formation *in vivo* has yet to be determined.

Here, we utilized 10× Genomics multiome reagents to create a detailed, time-resolved single-cell atlas of gene expression (RNA) and chromatin accessibility (assay for transposase-accessible chromatin [ATAC]) during murine hematovascular embryoid body (EB) differentiation. This approach identified robust production of both YS and allantois cell types. Using a combination of *in vitro* and *in vivo* assays, we demonstrate that Eomes is essential for the formation of YS-fated ExEM but is not required for specification of the allantois lineage and allantois endothelium. Eomes dependence thus clearly defines the formation of distinct extraembryonic hematovascular tissues. The previously described block in YS hematopoiesis, caused by Eomes loss of function, reflects an earlier loss in YS-fated ExEM. Moreover, we demonstrate that simultaneous disruption of Eomes and T entirely blocks the specification of all YS and allantois mesoderm. Taken together, these findings reveal new insights into the mechanisms that allow TFs to coordinate ExEM diversification. Additionally, the ability to readily generate allantoic mesoderm during embryonic stem cell (ESC) differentiation *in vitro* has important implications for future studies of developmental biology and regenerative medicine.

RESULTS

Efficient generation of ExEM subtypes *in vitro*

We recently described a mouse ESC-differentiation protocol that robustly generates extraembryonic populations, including YS-like hematopoietic progenitors, via the formation of EBs.[10] However, a comprehensive and unbiased analysis exploring how this *in vitro* system recapitulates *in vivo* embryonic development is missing. Here, we characterized the transcriptional/chromatin accessibility profiles of over 19,000 single cells, isolated at 12-h intervals from days 3 to 5 of EB differentiation (Figures 1B and S1A–S1D). We annotated 12 cell types (see STAR Methods) by integrating our *in vitro* dataset with an *in vivo* mouse gastrulation reference, performing label transfer (Figures S1E–S1G), and analyzing marker gene and motif accessibility patterns (Figures 1C–1E, S1H, and S1I).[17–19] Additionally, to aid motif interpretability, we predicted TF occupancy using *in silico* chromatin immunoprecipitation (ChIP) methodology[18] (Figures 1D and 1E; see STAR Methods).

At day 3, EBs were primarily composed of cells expressing PS markers including Nanog, Zic3, and Pou5f1 (Figures 1B, 1C, and S1E–S1I).[20] 12 h later, two early mesoderm (Early-Mes) cell populations emerged, which expressed mesodermal markers T, Eomes, Mixl1, Mesp1, and Lhx1 and primarily aligned with *in vivo* nascent mesoderm (Figures 1B, 1C, and S1E–S1I).[6,21–23] However, these populations showed distinctive molecular profiles (Table S1). The population expressing higher levels of Fgf8, Rspo3, and Igfbp3 was labeled Early-Mes #1, and the population expressing higher levels of Kdr, Pdgfra, and Nrp1, Early-Mes #2 (Figure 1C). Day 3.5 EBs also contained hematoendothelial (HE) precursors, expressing Early-Mes #2 markers as well as Kdr, Etv2, and Fgf10 (Figure 1C).[24–26]

Beginning at day 4, we detected further differentiated HE cells, which had downregulated mesodermal markers and expressed high levels of Etv2, Kdr, and Fli1, with regions of increased chromatin accessibility enriched for ETV2, GATA2, and ERG motifs (Figures 1B–1E).[24–26] During *in vivo* mouse gastrulation, Foxf1 and Dlk1 are broadly expressed in ExEM cells of the allantois, amnion, and chorion but excluded from hematopoietic cells in YS blood islands (Figures S1H and S1I),[27,28] while Pitx1, Tbx4, and Hoxa10/11/13 expression is localized to the allantois bud (Figures S1H and S1I).[28–30] Our day 4 EBs contained ExEM cell types that we labeled posterior mesoderm, mesenchyme, and allantois precursor based on the expression and/or motif accessibility patterns of Foxf1, Dlk1, Gata5/6, Tbx4, Pitx1, and Hoxa11 (Figures 1B–1E and S1E–S1I).

By days 4.5/5, EBs contained blood progenitors (Runx1, Hbb-bh1, Ikzf1, and RUNX1/GATA1/TAL1 motifs), endothelial cells (Cdh5, Tek, Fli1, and ETV2/SOX/ERG motifs),[17,19] mesenchyme, and an allantois cell population (Tbx4/Pitx1 and CDX/HOX/PITX1 motifs) (Figures 1B–1E).[3,28] We detected a few primordial germ cell (PGC)-like cells, expressing Gjb3 and Ifitm3, enriched for TFAP2C motifs, from day 3.5 onward (Figures 1B–1E).[31,32] Notably, the gene expression and chromatin landscapes of EB cell types closely mirrored their *in vivo* counterparts (Figures S1H, S1I, S2A, and S2B). Altogether, these findings demonstrate that our EB differentiation system effectively recapitulates the formation of ExEM cell populations that arise in the YS and allantois, including downstream primitive hematopoietic cells, endothelium, and mesenchymal cell types.

Multiome analysis identifies two distinct trajectories guiding the formation of the YS and allantois lineages

Next, we performed trajectory inference and identified two major trajectories (Figure 1F). Trajectory #1 generates allantois-like cells from posterior mesodermal precursors arising from Early-Mes #1, whereas trajectory #2 resembles YS formation from HE precursors arising from Kdr+ Early-Mes #2. Notably, the PS

Figure 1. Multiomic characterization of EBs identifies YS and allantois lineage differentiation

(A) Schematic of an E8.25 mouse gastrula. Ex, extraembryonic; Pr, proximal; A, anterior; P, posterior; D, distal; BI, blood islands.

(B) Uniform manifold approximation and projection (UMAP) of shared scRNA-seq and single-cell ATAC-seq (scATAC-seq), colored by cell type annotation (left) and day (right). D, day; Mes, mesoderm; PGC, primordial germ cell; HE, hematoendothelial.

(C–E) Molecular characterization of EB cell types, displaying marker gene expression (C), ChromVAR scores (D), and motif enrichment in ATAC marker peaks (E).

(F) Left-side, cell type densities displayed along pseudo-times for allantois trajectory #1 (top) and YS trajectory #2 (bottom). Right-side, pseudo-times for trajectory #1 (top) and trajectory #2 (bottom) overlaid on UMAPs. Cell types and days are colored as in (B).

(G) Heatmap showing normalized chromatin accessibility for peaks along pseudo-time for both trajectories, clustered by peak accessibility patterns. Enriched motifs for cluster(s) are displayed on the right. Differential testing was performed on pseudo-time range indicated in (F).

(H) Gene expression (solid lines) and chromVAR motif accessibility (dashed lines) for allantois (purple, trajectory 1) and YS (green, trajectory 2) trajectories plotted along pseudo-time.

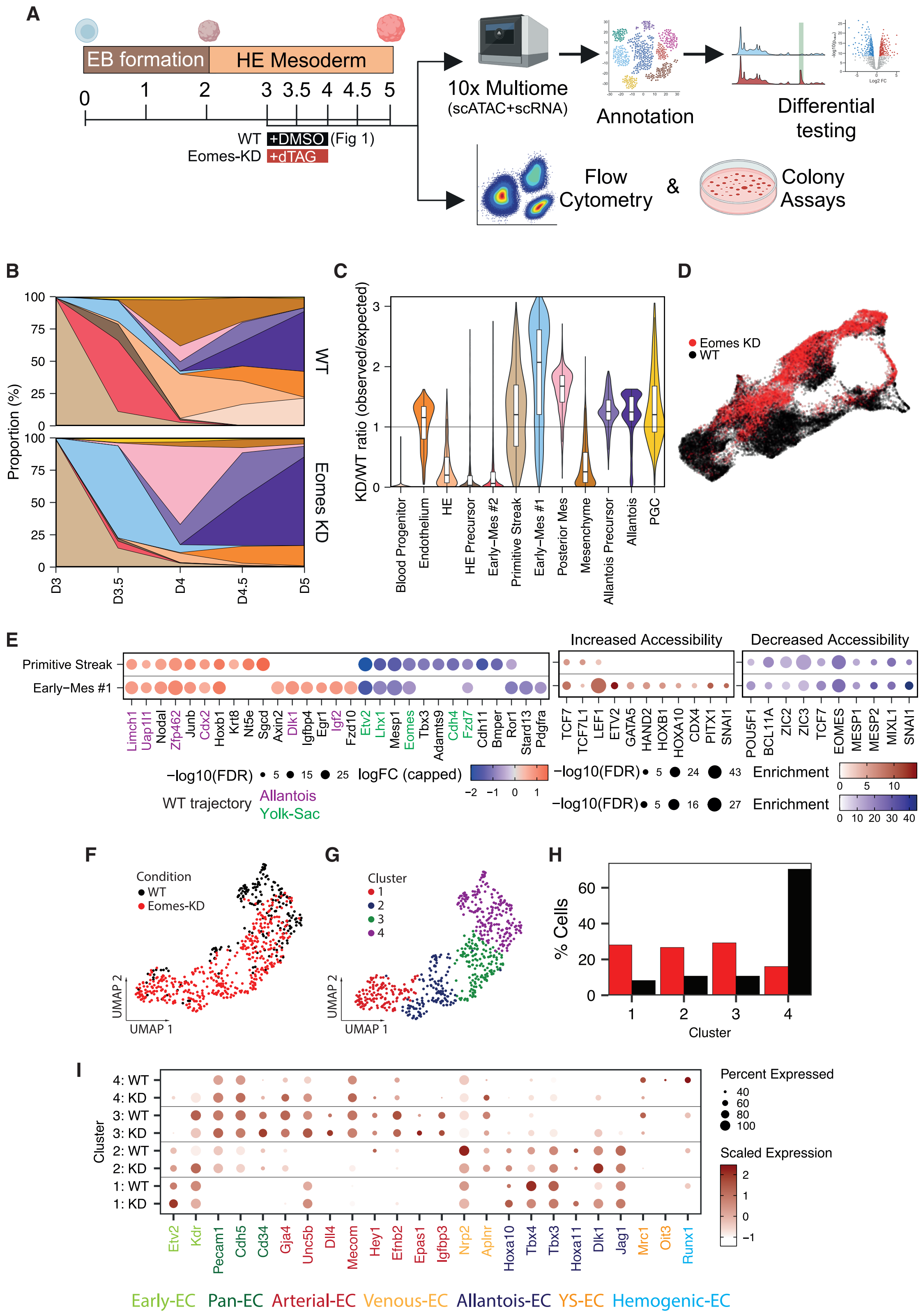

Figure 2. Allantois cell types predominate downstream of Eomes protein KD in EB cultures due to a disruption in Early-Mes formation

(A) Experimental design: 10× Genomics multiome workflow, flow cytometry, and colony assays were conducted on WT and Eomes-KD EB cultures. WT cultures received DMSO, and Eomes-KD cultures received dTAG13 from days 3 to 4.

(legend continued on next page)

and Early-Mes #1 are included in both trajectories prior to an early (day 3.5) lineage bifurcation (Figures 1B and 1F) and are therefore likely common precursors for both lineages, consistent with transplantation studies showing continuous generation of YS- and allantois-fated mesoderm from the posterior PS.[1]

Using TradeSeq, we identify gene sets and chromatin regions that display distinctive patterns of expression/accessibility across the pseudo-times for both trajectories (Figures 1G, 1H, S2C, and S2D; Table S1). Eomes, Lhx1, and Etv2 increased in expression and motif accessibility at the onset of the YS trajectory, whereas Snai2 and Hand1 were upregulated early in the allantois trajectory (Figures 1G and 1H). At later pseudo-times, Sry-box TFs, Sox7, and Sox6 exhibited specificity for the YS and allantois trajectories, respectively (Figure 1H). Signaling-related genes were also differentially expressed. Early in the YS trajectory, the ligand Vegfc and its receptor Kdr were upregulated (Figure S2D), consistent with lineage tracing experiments tracking Kdr+ mesoderm to YS blood islands.[33] Wnt and Fgf receptors/ligands (Wnt: Fzd7, Frzb, Wnt5a, and Lgr4; Fgf: Fgf10/15/18) as well as transforming growth factor β (Tgf-β)/BMP pathway elements, including receptors (Tdgf1 and Tgfbr3), inhibitors (Lefty2 and Bambi), ligands (Bmp2, Bmp7, and Tgfb2), and downstream effectors (Smad9), also exhibit distinctive expression patterns (Figure S2D). Overall, our analyses identify dynamic and distinctive expression patterns of inductive cues and TFs, which potentially direct cell fate specification at various stages of YS and allantois differentiation.

Eomes expression in ExEM progenitors is required for the YS lineage but dispensable for allantois cell populations

Using Eomes knockout (KO) ESC, we previously identified an essential role for Eomes in YS-like hematopoiesis, with chromatin changes of day 4 KDR+ cells suggesting that Eomes primes Early-Mes with hemogenic competence.[10] The trajectory analyses presented above suggest Eomes acts at the onset of ExEM diversification (days 3–3.5), when bifurcation toward the YS versus allantois lineage occurs (Figure 1H). To further investigate Eomes requirements during this early time window, we employed flow cytometry, functional assays, and multiomics using a Runx1^{Venus} Eomes$^{mCherry\text{-}degron}$ ESC line (Figure 2A).[34] This line features a degron tag attached to the C terminus of Eomes, which enables rapid EOMES protein depletion by adding the small molecule dTAG13 to culture media.[34] Additionally, the Venus reporter in this line monitors Runx1 expression, which marks nascent hematopoietic progenitors.[35]

At day 3 of EB differentiation, flow cytometry shows ~70% of cells expressed EOMES protein prior to upregulation of mesodermal markers KDR and PDGFRA (Figures S3A and S3B). The proportion of EOMES-expressing cells dropped substantially by day 4 (~15%), and by day 4.5 EOMES was no longer detectable (Figure S3A). Addition of dTAG13 from days 3 to 4, hereafter referred to as Eomes knockdown (Eomes-KD), rapidly abolished EOMES protein and blocked formation of a day 3.5 KDR$^+$PDGFRA$^+$ population (Figures S3A and S3B). In line with previous Eomes-KO experiments,[10] Eomes-KD abrogated formation of Runx1-Venus+/CD41+ blood progenitors and primitive erythrocytes but failed to block generation of CDH5+ endothelial cells (Figures S3C and S3D). Kinetics of RNA versus protein expression for these markers were highly similar during wild-type (WT) EB differentiation (Figures S3A, S3B, S3D, and S3E).

To further investigate the molecular consequences of Eomes-KD, we conducted multiomic profiling of Eomes-KD EBs at 12-h intervals from days 3.5 to 5 and compared these profiles to equivalent DMSO-treated WT EBs shown in Figure 1 (Figure 2A). Strikingly, Eomes-KD cells display a markedly reduced ability to contribute to cell types of the YS trajectory (Figures 2B–2D). By day 3.5, Eomes-KD cultures produced far fewer Early-Mes #2 and HE precursors compared with WT cultures, with later stages showing a notable reduction in HE and blood progenitors. By contrast, cell types associated with the allantois trajectory, including Early-Mes #1, posterior mesoderm, allantois precursors, and allantois populations, were abundantly generated (Figures 2B–2D). These results demonstrate that Eomes governs the formation of an early subset of YS-fated ExEM and its downstream progeny but is dispensable for allantois formation.

To further explore Eomes' role during the initial step of ExEM diversification, we examined Eomes-dependent molecular changes in PS and Early-Mes #1 cell types, the relatively undifferentiated common precursors for both trajectories (Figure 2E; Table S1). Notably, Eomes disruption caused downregulation of Etv2, a key regulator of YS hematovascular formation,[26,36–38] as well as Eomes target genes Lhx1, Mixl1, and Mesp1[9,10,39] (Figure 2E). Additionally, motifs for EOMES, mesoderm-related TFs (MESP1/2, MIXL1, and SNAI1), and pluripotency factors (ZIC2/3 and POU5F1) were enriched in regions with decreased chromatin accessibility (Figure 2E). Conversely, Eomes-KD led to premature upregulation of TFs and signaling molecules (Cdx2, Nodal, Dlk1, and Igf2) (Figures 2E and S3F) as well as increased chromatin accessibility in regions enriched for motifs (HOX, CDX4, and PITX1) linked to later stages of the allantois trajectory (Figures 1D, 1E, and 1G). Interestingly, Eomes expression was also reduced, implying an autoregulatory mechanism likely drives Eomes expression during YS formation (Figure 2E). Thus, Eomes controls chromatin and transcriptional biases during ExEM diversification that pattern allantois versus YS fates in an early posterior PS population.

(B) Cell type proportions per time point for WT (top) and Eomes-KD cultures (bottom). At day 3 WT proportions are displayed, as Eomes-KD was induced from day 3 onward. Cell type colored as in Figure 1B.
(C) Ratio of Eomes-KD to WT cells, calculated using the 100 nearest neighbors for each cell, colored by cell type. Values above one indicate enrichment of Eomes-KD cells, while values below one indicate depletion.
(D) UMAPs colored by EB culture condition showing Eomes-KD cells in red and WT cells in black.
(E) Left panel shows differential gene expression between Eomes-KD and WT early cell types. Colored gene names indicate allantois (purple) and YS (green) trajectory marker genes from Figure 1. Middle/right panels display motif enrichment for peaks with increased (middle) or decreased (right) chromatin accessibility.
(F and G) Detailed analysis of endothelium displaying UMAP colored by EB culture condition (F) or sub-cluster (G).
(H) Proportion of cells in each of the endothelial subclusters, colored by EB culture condition.
(I) Gene expression dotplot of endothelial marker genes for each of the endothelial subclusters and EB culture conditions.

Eomes depletion promotes allantois endothelium production *in vitro*

The YS and allantois are primary sites of *de novo* vasculogenesis during embryogenesis, distinguished by specific endothelial molecular signatures.[17,19] YS endothelium is marked by Lyve1, Stab2, Oit3, and Mrc1, whereas developing endothelium of the allantois expresses Dlk1, Jag1, Hoxa10/11, and Tbx4.[19] At days 4.5/5, Eomes-KD and WT EBs contained similar proportions of developing endothelial cells (Figure 2C). To investigate endothelial heterogeneity, we sub-clustered Eomes-KD and WT endothelium and explored the expression of arterial, venous,[40] and YS versus allantois endothelial marker genes (Figures 2F–2I).

Eomes-KD EBs abundantly generated endothelial precursors with an allantois signature (Figures 2F–2I, clusters 1 and 2), characterized by high expression of markers such as Kdr, Etv2, Hoxa10/11, Tbx4, Dlk1, and Jag1, which were less prominent in WT EBs. Both Eomes-KD and WT EBs contained more differentiated endothelial cells, including cells expressing arterial markers (Unc5b, Dll4, Efnb2, etc.—cluster 3). However, cluster 4 endothelial cells differed between conditions: WT EBs abundantly generated these cells, which expressed some YS endothelial markers (Mrc1 and Oit3) and the hemogenic marker Runx1. In contrast, Eomes-KD EBs produced cluster 4 endothelial cells, which displayed reduced YS-specific marker expression. These results suggest Eomes-KD EBs preferentially generate endothelium via an allantois differentiation pathway.

Chimeric embryo analyses validate critical functions of Eomes for YS but not allantois formation

To investigate the predictive power of our EB model and further explore a role for Eomes during YS and allantois formation *in vivo*, we conducted single-cell RNA sequencing (scRNA-seq) on 4 pools of chimeric embryos generated by injecting WT blastocysts with 4 independent Eomes-KO ESC clones expressing TdTomato (Figures 3A and S3F). At E8.25–E8.5, chimeric embryo pools were dissociated, WT host cells and TdTomato+ Eomes-KO cells were sorted using fluorescence-activated cell sorting (FACS) and analyzed by 10× Genomics scRNA-seq (Figure 3A). The transcriptional profiles of WT and Eomes-KO cells from chimeric embryos were aligned with a gastrulation reference atlas,[17] enabling cell type annotation and identification of cellular and molecular disruptions caused by Eomes-KO (Figures 3B and S3G).

WT cells successfully mapped to the ectodermal, mesodermal, and endodermal cell types typically found in E8.25 embryos, as well as non-epiblast-derived lineages such as extraembryonic endoderm and ectoderm (Figure 3B). By contrast, Eomes-KO cells fail to form non-epiblast-derived lineages, reflecting the restricted potency of ESC (Figure 3B). To investigate statistical differences in the contribution of WT versus Eomes-KO cells to various cellular populations, we performed neighborhood-level differential abundance testing (Figures 3C and S3H).[41] Consistent with previous fate mapping and functional studies,[8–10] Eomes-KO cells were deficient in generating gut tube and anterior mesodermal derivatives, such as cranial and pharyngeal mesoderm and cardiomyocytes (Figures 3B and 3C). However, Eomes-KO cells efficiently contribute to posterior mesodermal derivatives, including caudal and somitic mesoderm, although formation of paraxial mesoderm was impaired (Figures 3B and 3C).

Notably, Eomes-KO cells failed to form YS endothelium and blood progenitors but were able to generate allantois and allantois endothelial cells (Figures 3B and 3C), aligning with the block in YS differentiation observed in our EB model system. The transcriptional profiles of Eomes-KO versus WT allantois endothelium were highly concordant (Figure S3I; Table S2); however, numerous genes were differentially expressed in allantois and lateral plate mesoderm cell populations (Figure S3I; Table S2). Finally, to corroborate our chimera-seq findings, we additionally performed whole-mount immunofluorescence confocal imaging of Eomes-KO chimeric embryos, which confirmed a complete absence of tdTomato+ Eomes-KO cells in the YS but a significant contribution to the allantois, including Pecam1+ allantois endothelium (Figures 3D and S4A; Video S1).

Eomes and T play distinct and compensatory roles during ExEM formation

During gastrulation, T expression begins shortly after Eomes in the proximal posterior epiblast and the nascent mesoderm along the length of the PS.[10] It is subsequently observed in various mesodermal derivatives, such as the cell population at the base of the allantoic bud, including the PGCs,[42] as well as in the notochord and neuro-mesodermal progenitors (Figure 4A).[5,7,11,17] We previously conducted scRNA-seq on T-KO chimeric embryos at E8.5.[14] Reanalysis of these data identifies no evident disruption of YS blood and endothelium, cells, or allantois cell types, including allantois endothelium (Figure 4B). These findings are consistent with earlier studies that show T-deficient ESC can contribute to the developing allantois region in chimeric embryos at E8.25.[16] Chorio-allantois fusion, which occurs at slightly later developmental stages, is impaired in highly chimeric T-deficient allantoizes, mimicking T-null embryos.[15,43]

Several ExEM populations, including the allantois, allantois endothelium, and the lateral plate mesoderm, form successfully in both Eomes-KO and T-KO chimeric embryos (Figures 3C and 4B). Interestingly, Eomes-KO alters the transcriptomes of allantois and lateral plate mesoderm cells, while equivalent cells in T-KO chimeras remain largely unaffected (Figure 4C). The formation and molecular characteristics of Eomes-KO and T-KO allantois endothelium, however, are highly similar and not disrupted (Figure 4C). Therefore, disrupting T alone does not impact the formation or the transcriptional properties of YS or allantois cell types. Rather, neuro-mesodermal progenitors (NMPs) are the major cell type transcriptionally dysregulated in T-KO chimeras (Figures 4B and 4C). By contrast, Eomes is essential for the formation of YS cell types, and, while Eomes-KO allantois cells are specified, they exhibit aberrant gene expression profiles that may impact downstream functional properties.

Disrupting both Eomes and T simultaneously completely blocks intra-embryonic mesoderm formation in ESC models[12,13]; however, the impact on specification of ExEM lineages has not been formally explored. In line with our transcriptional analyses of single KO chimeras, imaging confirms that T-KO tdTomato+ and Eomes-KO tdTomato+ ESC contributed to the allantois region in E8.25 chimeric embryos, including Pecam1+ allantoic endothelium (Figures 3D, 4D, S4A–S4C, and S4E; Videos S2A and S2B). In striking contrast, Eomes/T double-KO (dKO) tdTomato+ ESC were absent from both the allantois and YS,

CellPress
OPEN ACCESS

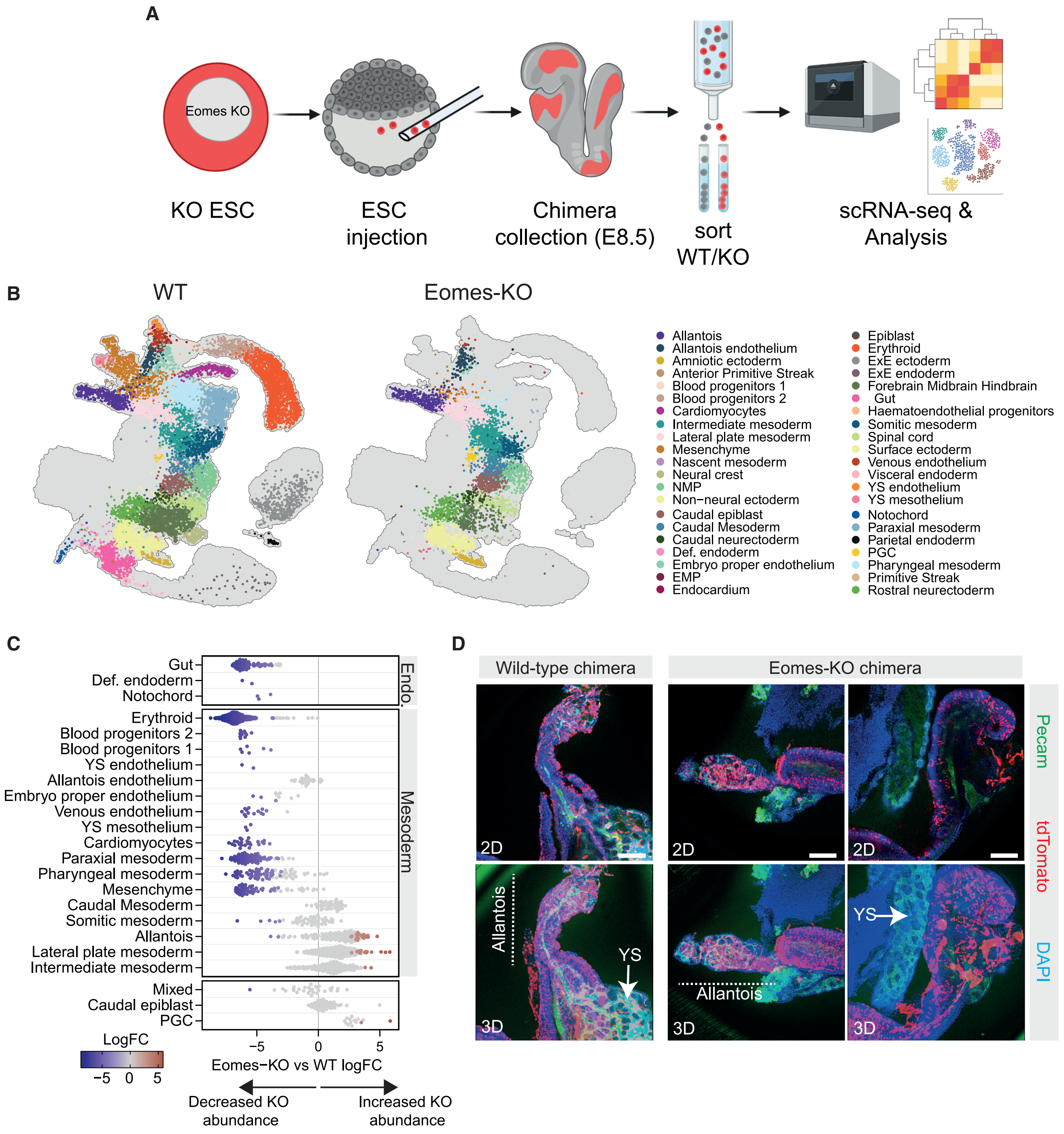

Figure 3. Eomes is essential for YS but dispensable for allantois development *in vivo*

(A) Experimental design for Eomes-KO/WT chimaera-seq. Created with BioRender.com.

(B) Projection of chimera-seq WT (left) and Eomes-KO (right) cells onto the gastrulation atlas UMAP from Pijuan-Sala et al.,[17] colored by cell type annotation.

(C) Neighborhood-level differential abundance testing between Eomes-KO and WT cells. Negative logFC values indicate depletion of KO cells, while positive logFC values indicate enrichment, and neighborhoods without statistical significance are colored gray.

(D) Confocal imaging of chimeras generated by injecting WT (left) or Eomes-KO (right) tdTomato+ ESCs into WT blastocysts. Samples are stained for DAPI (blue), injected tdTomato+ cells (red), and Pecam-1 (green). YS and allantois structures are indicated. Scale bars: 200 μm.

indicating a complete block in the ability of Eomes/T dKO ESC to form cell types of these extraembryonic tissues (Figure 4D; Videos S2A and S2B). Furthermore, depleting Eomes protein in Runx1^{Venus} Eomes$^{mCherry\text{-}degron}$ T-KO EBs blocked the production of KDR+ and/or PDGFRA+ mesodermal cell populations (Figures 4E and S4B–S4D). Together, these findings demonstrate

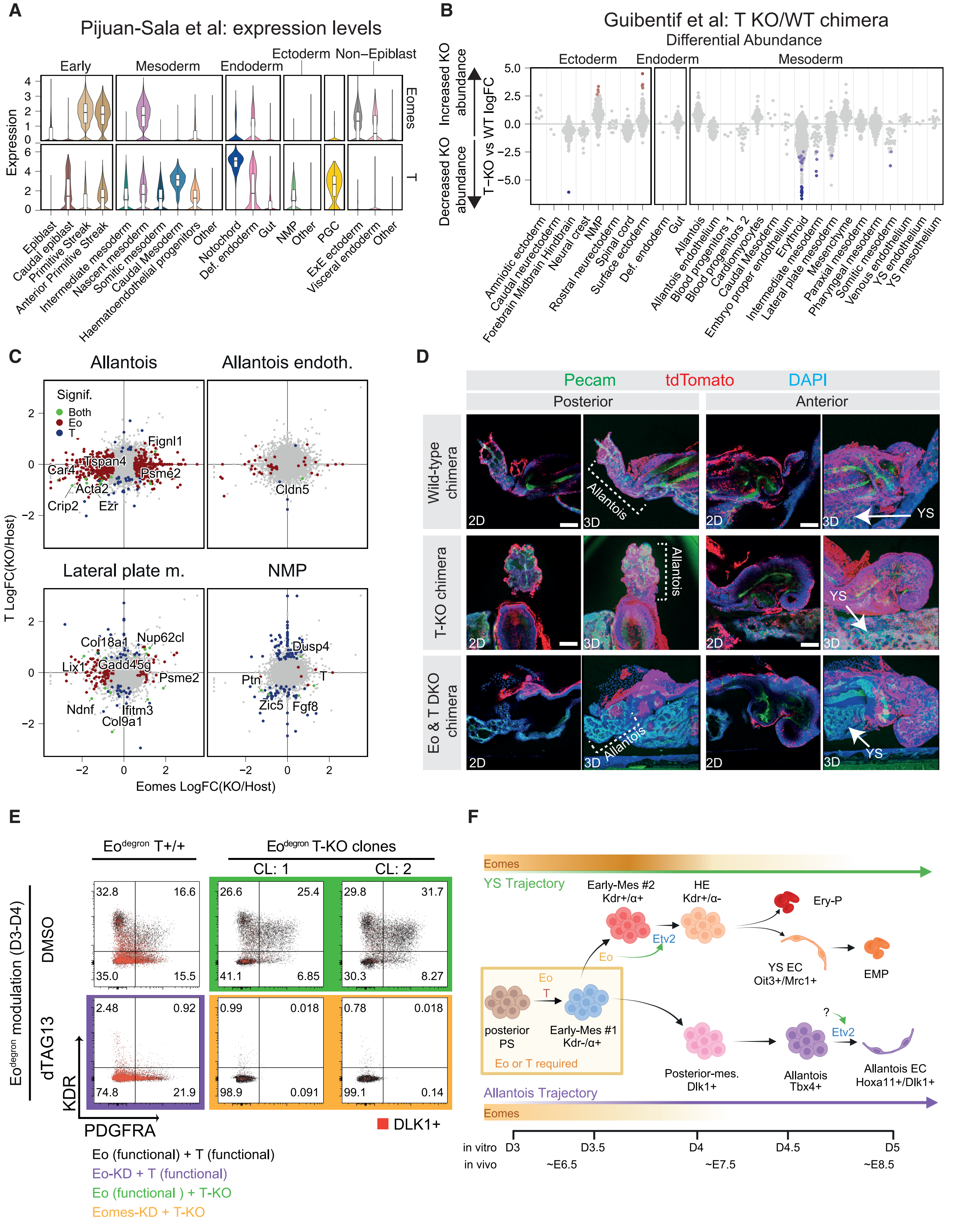

Figure 4. Eomes and T have distinct and compensatory roles during ExEM formation

(A) Violin plots displaying Eomes (top) and T (bottom) RNA expression patterns from the Pijuan-Sala et al.,[17] gastrulation atlas, which covers E6.5–E8.5 of mouse development. Cell types with low expression are relabeled as “other.” Def. endoderm, definitive endoderm; NMP, neuro-mesodermal progenitor.

(legend continued on next page)

that while individual KO of Eomes or T does not disrupt the initial generation of allantois mesodermal cells, simultaneous disruption of both T-box factors does.

To further explore the interplay between Eomes and T, we compared the Eomes-KD and WT molecular profiles along allantois differentiation in our *in vitro* multiomics dataset. Notably, in the absence of Eomes, T RNA expression is upregulated during early allantois differentiation (Figure S4F; Table S1). Additionally, we identified a cluster of chromatin regions that exhibit increased accessibility upon Eomes-KD, of which approximately 60% are bound by T in a previously published Eomes/T ChIP-seq dataset from Tosic et al.[13] (Figure S4G, cluster 3; Table S1). These analyses highlight putative T-bound chromatin regions that may compensate for loss of Eomes and facilitate allantois differentiation, providing insights for future studies into this regulatory interplay.

DISCUSSION

The specification and diversification of ExEM lineages are crucial for the growth and development of the mammalian embryo within the uterine environment. Here, we show a robust EB model mimics temporal induction of ExEM subtypes that arise during gastrulation, generating cell populations that adopt distinct YS or allantoic ExEM fates. Using this system, we identify TFs, signaling pathways, and chromatin accessibility changes associated with the emergence of two major ExEM trajectories. Additionally, we identify a mesenchyme population that potentially represents precursors of two additional ExEM lineages, the amnion and chorion. Our model, therefore, highlights unexpected mesodermal and endothelial diversity during EB differentiation, thus enabling a more precise interpretation of stem cell differentiation pathways, which is essential for downstream therapeutic applications.

Using a degron-based approach in our EB model, alongside validation experiments in chimeric embryos, we identify a seminal role for Eomes in directing specification of YS but not allantoic fates. Eomes depletion induces premature activation of TFs and signaling pathways associated with later stages of allantois formation, suggesting Eomes likely represses allantoic ExEM specification in the early posterior PS when YS formation predominates. Notably, our work identifies a downstream network of hematovascular TFs that Eomes activates, including Mixl1, Mesp1, and the key regulator Etv2, in agreement with a recent study showing Etv2 upregulation upon Eomes overexpression in a similar EB model system.[44] Etv2 is an upstream component of the transcriptional hierarchy regulating induction of hematovascular fate in the embryo proper, YS, and allantois.[26,36–38] Here, we show that Eomes controls Etv2 expression at the onset of YS ExEM specification prior to KDR expression, but not during allantois endothelial formation (Figure 4F). Therefore, we demonstrate that distinct regulatory mechanisms drive Etv2 expression in diverse ExEM lineages and place Eomes at the top of a transcriptional hierarchy specifically regulating YS hematovascular formation.

The T-box family TFs Eomes and T exhibit distinctive expression patterns during murine gastrulation. Eomes is transiently expressed in ExEM precursors that traverse the posterior PS starting at E6.5, while T appears slightly later throughout the PS and nascent mesoderm.[10] Unlike Eomes, which is crucial for cardiac[9] and YS mesoderm specification, T directs mesodermal formation from NMPs, facilitating posterior axis extension.[14–16] Our chimera studies show that neither TF individually has roles in the initial formation of the allantois mesoderm. Previous studies have shown that disrupting both TFs completely blocks intraembryonic mesoderm formation, pointing to partial redundancy.[12,13] Importantly, our study demonstrates similar redundancy applying to the extraembryonic compartment, since elimination of both Eomes and T ablates ExEM formation both *in vitro* and *in vivo*. Our integrative analysis with ChIP-seq datasets suggests that increased T activity upon Eomes loss-of-function compensates for allantois mesoderm specification.

Altogether, our findings suggest Eomes and T likely act in a balancing mechanism to control YS versus allantois mesoderm specification as the PS elongates and their expression patterns shift during gastrulation. While this study sheds light on their partially redundant roles, further research is needed to fully elucidate the molecular mechanisms underpinning this balance. Importantly, the comprehensive datasets and EB model we provide offer a robust foundation for further investigations into the genetic networks that specify and pattern extraembryonic tissues—an often overlooked but crucial aspect of embryogenesis.

Limitations of the study

We delineate the molecular dynamics of YS and allantois differentiation using *in vitro* datasets; however, direct comparison to *in vivo* references is constrained by limited single-cell data for

(B) Neighborhood-level differential abundance testing between T-KO and WT cells. Negative logFC values indicate depletion of KO cells, while positive logFC values indicate enrichment, and neighborhoods without statistical significance are colored gray. YS, yolk sac; Def. endoderm, definitive endoderm; NMP, neuromesodermal progenitor.

(C) Comparison of DEGs between injected KO and host WT cells for Eomes (*x* axis) and T (*y* axis) chimera-seq experiments within specific cell types. Highlighted genes are significant in either T (blue), Eomes (red), or both KO conditions (green).

(D) Confocal imaging of chimeras generated by injecting WT (row 1), T-KO (row 2), Eomes/T dKO (row 3), and TdTomato+ ESC into a WT host blastocyst. Samples are stained for DAPI (blue), injected tdTomato+ cells (red), and Pecam-1 (green). YS and allantois structures are indicated. Scale bars, 200 μm.

(E) Flow cytometric analyses of PDGFRA and KDR expression for $Eomes^{WT}/T^{WT}$, $Eomes^{KD}/T^{WT}$, $Eomes^{WT}/T^{KO}$, and $Eomes^{KD}/T^{KO}$ at day 4 of EB differentiation. Red dots indicate DLK1+ cells.

(F) Model summarizing molecular regulation of YS and allantois mesodermal lineage specification. Eomes plays a crucial role in differentiation toward the YS mesoderm fate, causing Eomes loss-of-function cells to be redirected toward the allantois lineage. Etv2, required for hematovascular differentiation, is controlled by Eomes during YS formation but not during the formation of allantois endothelium. The simultaneous KO of Eomes and T, but not their individual KOs, completely ablates the formation of ExEM. Orange bars indicate Eomes expression levels along differentiation trajectories. α, Pdgfra; EC, endothelial cell; YS, yolk sac; PS, primitive streak; Ery-P, primitive erythroid; HE, hematoendothelium; Posterior-Mes, posterior mesoderm; Early-Mes, early mesoderm; EMP, erythromyeloid progenitor.

this developmental window and ground truth lineage information. The Eomes and T ChIP-seq data in Figure S4G, generated in a different *in vitro* system,[13] may not fully capture their chromatin binding dynamics during ExEM differentiation.

RESOURCE AVAILABILITY

Lead contact

Further information and requests for resources should be directed to and will be fulfilled by the lead contact, Berthold Gottgens (bg200@cam.ac.uk).

Materials availability

Cell lines and other reagents generated in this study are available upon request from the lead contact, and other materials in this study are commercially available.

Data and code availability

- Data from this study have been deposited at the Gene Expression Omnibus (GEO: GSE274166 and GEO: GSE274167). Chromatin accessibility profiles can be explored using the UCSC Genome Browser at https://genome.ucsc.edu/s/Barttheeuwes/Theeuwes_Harland_vitro_multiome. Previously published T-KO chimera scRNA-seq was reanalyzed and can be found on Arrayexpress under code E-MTAB-8811 or accessed via the R package "MouseGastrulationData" via the "TChimeraData" function. Reference atlas data used throughout the paper can be accessed at https://marionilab.github.io/ExtendedMouseAtlas/ for scRNA-seq[17,19] and GEO: GSE205117 for the multiome gastrulation atlas.[18] Previously published T and Eomes ChIP-seq data can be found at GEO: GSE128466.[13]
- All code used to process and analyze the data is available at https://github.com/BartTheeuwes/Eomes_ExEM.
- Any additional information required to reanalyze the data reported in this paper is available from the lead contact upon request.

ACKNOWLEDGMENTS

We thank William Mansfield at the CRUK Pre-clinical Genome Editing facility for generating embryos for the chimera-seq experiments, the Flow Cytometry Core Facility at CIMR for cell sorting, CRUK-CI genomics core for preparing the single-cell libraries and sequencing of chimera-seq experiments, the Babraham Institute's Genomics Facility for sequencing of 10× multiome experiments, Darran Clements from the CSCI Imaging Facility for microscopy support, Rebecca Hannah for data support, and the Cambridge Research Computing Services for supplying computer resources. We also thank the Dunn School Don Mason Flow Cytometry Facility, the Dunn School Bioimaging Facility, Genome Engineering Oxford, and the Pathology Support Building Facility. Finally, we would like to thank all members of the Robertson and Gottgens labs for their advice and support, including Philipp Kurbel and Atreyi Biswas for technical assistance. Work at Cambridge was supported by the Wellcome Trust (Wellcome Collaborative Gastrulation Consortium Award, 220379/B/20/Z B.G.; Wellcome Early-Career Award, 226309/Z/22/Z L.T.G.H.; Wellcome PhD studentship, 224928/Z/22/Z B.T.). Work in the Gottgens group was also supported by Blood Cancer UK, the Medical Research Council, Cancer Research UK, and by core support grants from the Wellcome Trust (203151/Z/16/Z and 203151/A/16/Z) and the UKRI Medical Research Council (MC_PC_17230). Work at Oxford was supported by the Wellcome Trust (214175/Z/18/Z E.J.R. and 219978/Z/19/Z A.M.B.). E.J.R. is a Wellcome Trust Principal Fellow. For the purpose of open access, the author has applied a CC BY public copyright license to any author-accepted manuscript version arising from this submission.

AUTHOR CONTRIBUTIONS

Conceptualization and research design, L.T.G.H., B.T., E.J.R., and B.G.; EB differentiation experiments, L.T.G.H. and A.M.B.; bioinformatic analyses, B.T.; ESC cell-line generation, L.T.G.H., A.M.B., and E.J.R.; 10× Genomics multiomic sample processing, T.L. and S.J.C.; chimera injections and dissections, M.-L.N.T., E.J.R., and I.C.; imaging, I.C.; project support, N.K.W. and E.B.; data curation, B.T. and R.A.; writing, L.T.G.H., B.T., B.G., and E.J.R. wrote the manuscript with feedback from co-authors; supervision, E.J.R. and B.G.; project administration, L.T.G.H., E.J.R., B.G., and W.R.; and funding acquisition, L.T.G.H., B.T., B.G., E.J.R., and A.M.B.

DECLARATION OF INTERESTS

W.R., S.J.C., and R.A. have been employees of Altos Labs since September 2022.

DECLARATION OF GENERATIVE AI AND AI-ASSISTED TECHNOLOGIES IN THE WRITING PROCESS

During the preparation of this work, the author(s) used ChatGPT in order to improve clarity of writing. After using this tool/service, the author(s) reviewed and edited the content as needed and take(s) full responsibility for the content of the publication.

STAR★METHODS

Detailed methods are provided in the online version of this paper and include the following:

- KEY RESOURCES TABLE
- EXPERIMENTAL MODELS AND STUDY PARTICIPANTS
  - ESC genetic modification using CRISPR-Cas9
  - ESC maintenance
  - In vitro hematovascular EB differentiation protocol
  - Chimera generation
- METHOD DETAILS
  - Western blot
  - Flow cytometry
  - Collecting EB samples for 10x multiomics and flow cytometry
  - Nuclear extraction and 10x genomics multiome library preparation
  - *In vitro* RNA+ATAC Multiome pre-processing
  - *In vitro* RNA+ATAC Multiome wild-type analysis
  - *In vitro* RNA+ATAC Multiome Eomes knock-down analysis
  - Chimera imaging
  - Chimera-seq analysis
- QUANTIFICATION AND STATISTICAL ANALYSIS

SUPPLEMENTAL INFORMATION

Supplemental information can be found online at https://doi.org/10.1016/j.devcel.2025.06.001.

Received: August 13, 2024
Revised: February 10, 2025
Accepted: June 2, 2025
Published: June 24, 2025

REFERENCES

1. Kinder, S.J., Tsang, T.E., Quinlan, G.A., Hadjantonakis, A.K., Nagy, A., and Tam, P.P.L. (1999). The orderly allocation of mesodermal cells to the extraembryonic structures and the anteroposterior axis during gastrulation of the mouse embryo. Development *126*, 4691–4701. https://doi.org/10.1242/dev.126.21.4691.
2. Dzierzak, E., and Bigas, A. (2018). Blood Development: Hematopoietic Stem Cell Dependence and Independence. Cell Stem Cell *22*, 639–651. https://doi.org/10.1016/j.stem.2018.04.015.
3. Arora, R., and Papaioannou, V.E. (2012). The murine allantois: A model system for the study of blood vessel formation. Blood *120*, 2562–2572. https://doi.org/10.1182/blood-2012-03-390070.

CellPress
OPEN ACCESS

4. Downs, K.M. (2022). The mouse allantois: new insights at the embryonic-extraembryonic interface. Philos. Trans. R. Soc. Lond. B Biol. Sci. *377*, 20210251. https://doi.org/10.1098/rstb.2021.0251.

5. Inman, K.E., and Downs, K.M. (2007). The murine allantois: Emerging paradigms in development of the mammalian umbilical cord and its relation to the fetus. Genesis *45*, 237–258. https://doi.org/10.1002/dvg.20281.

6. Ciruna, B.G., and Rossant, J. (1999). Expression of the T-box gene Eomesodermin during early mouse development. Mech. Dev. *81*, 199–203. https://doi.org/10.1016/S0925-4773(98)00243-3.

7. Rivera-Pérez, J.A., and Magnuson, T. (2005). Primitive streak formation in mice is preceded by localized activation of Brachyury and Wnt3. Dev. Biol. *288*, 363–371. https://doi.org/10.1016/j.ydbio.2005.09.012.

8. Arnold, S.J., Hofmann, U.K., Bikoff, E.K., and Robertson, E.J. (2008). Pivotal roles for eomesodermin during axis formation, epithelium-to-mesenchyme transition and endoderm specification in the mouse. Development *135*, 501–511. https://doi.org/10.1242/dev.014357.

9. Costello, I., Pimeisl, I.M., Dräger, S., Bikoff, E.K., Robertson, E.J., and Arnold, S.J. (2011). The T-box transcription factor Eomesodermin acts upstream of Mesp1 to specify cardiac mesoderm during mouse gastrulation. Nat. Cell Biol. *13*, 1084–1091. https://doi.org/10.1038/ncb2304.

10. Harland, L.T.G., Simon, C.S., Senft, A.D., Costello, I., Greder, L., Imaz-Rosshandler, I., Göttgens, B., Marioni, J.C., Bikoff, E.K., Porcher, C., et al. (2021). The T-box transcription factor Eomesodermin governs haemogenic competence of yolk sac mesodermal progenitors. Nat. Cell Biol. *23*, 61–74. https://doi.org/10.1038/s41556-020-00611-8.

11. Herrmann, B.G., Labeit, S., Poustka, A., King, T.R., and Lehrach, H. (1990). Cloning of the T gene required in mesoderm formation in the mouse. Nature *343*, 617–622. https://doi.org/10.1038/343617a0.

12. Schüle, K.M., Weckerle, J., Probst, S., Wehmeyer, A.E., Zissel, L., Schröder, C.M., Tekman, M., Kim, G.-J., Schlägl, I.-M., Sagar, et al. (2023). Eomes restricts Brachyury functions at the onset of mouse gastrulation. Dev. Cell *58*, 1627–1642.e7. https://doi.org/10.1016/j.devcel.2023.07.023.

13. Tosic, J., Kim, G.-J., Pavlovic, M., Schröder, C.M., Mersiowsky, S.-L., Barg, M., Hofherr, A., Probst, S., Köttgen, M., Hein, L., et al. (2019). Eomes and Brachyury control pluripotency exit and germ-layer segregation by changing the chromatin state. Nat. Cell Biol. *21*, 1518–1531. https://doi.org/10.1038/s41556-019-0423-1.

14. Guibentif, C., Griffiths, J.A., Imaz-Rosshandler, I., Ghazanfar, S., Nichols, J., Wilson, V., Göttgens, B., and Marioni, J.C. (2021). Diverse Routes toward Early Somites in the Mouse Embryo. Dev. Cell *56*, 141–153.e6. https://doi.org/10.1016/j.devcel.2020.11.013.

15. Wilson, V., Rashbass, P., and Beddington, R.S.P. (1993). Chimeric analysis of *T (Brachyury)* gene function. Development *117*, 1321–1331. https://doi.org/10.1242/dev.117.4.1321.

16. Rashbass, P., Cooke, L.A., Herrmann, B.G., and Beddington, R.S.P. (1991). A cell autonomous function of Brachyury in T/T embryonic stem cell chimaeras. Nature *353*, 348–351. https://doi.org/10.1038/353348a0.

17. Pijuan-Sala, B., Griffiths, J.A., Guibentif, C., Hiscock, T.W., Jawaid, W., Calero-Nieto, F.J., Mulas, C., Ibarra-Soria, X., Tyser, R.C.V., Ho, D.L.L., et al. (2019). A single-cell molecular map of mouse gastrulation and early organogenesis. Nature *566*, 490–495. https://doi.org/10.1038/s41586-019-0933-9.

18. Argelaguet, R., Lohoff, T., Li, J.G., Nakhuda, A., Drage, D., Krueger, F., Velten, L., Clark, S.J., and Reik, W. (2022). Decoding gene regulation in the mouse embryo using single-cell multi-omics. Preprint at bioRxiv. https://doi.org/10.1101/2022.06.15.496239.

19. Imaz-Rosshandler, I., Rode, C., Guibentif, C., Harland, L.T.G., Ton, M.-L. N., Dhapola, P., Keitley, D., Argelaguet, R., Calero-Nieto, F.J., Nichols, J., et al. (2024). Tracking early mammalian organogenesis – prediction and validation of differentiation trajectories at whole organism scale. Development *151*, 1–17. https://doi.org/10.1242/dev.201867.

20. Elms, P., Scurry, A., Davies, J., Willoughby, C., Hacker, T., Bogani, D., and Arkell, R. (2004). Overlapping and distinct expression domains of Zic2 and Zic3 during mouse gastrulation. Gene Expr. Patterns *4*, 505–511. https://doi.org/10.1016/j.modgep.2004.03.003.

21. Wolfe, A.D., and Downs, K.M. (2014). Mixl1 localizes to putative axial stem cell reservoirs and their posterior descendants in the mouse embryo. Gene Expr. Patterns *15*, 8–20. https://doi.org/10.1016/j.gep.2014.02.002.

22. Perea-Gómez, A., Shawlot, W., Sasaki, H., Behringer, R.R., and Ang, S.L. (1999). HNF3β and Lim1 interact in the visceral endoderm to regulate primitive streak formation and anterior-posterior polarity in the mouse embryo. Development *126*, 4499–4511. https://doi.org/10.1242/dev.126.20.4499.

23. Probst, S., Tosic, J., Schwan, C., Grün, D., and Arnold, S.J. (2021). Spatiotemporal sequence of mesoderm and endoderm lineage segregation during mouse gastrulation. Development *148*, dev193789. https://doi.org/10.1242/dev.193789.

24. Yamaguchi, T.P., Dumont, D.J., Conlon, R.A., Breitman, M.L., and Rossant, J. (1993). Flk-1, an Flt-Related Receptor Tyrosine Kinase Is an Early Marker for Endothelial Cell Precursors. Development *118*, 489–498. https://doi.org/10.1242/dev.118.2.489.

25. Kataoka, H., Hayashi, M., Nakagawa, R., Tanaka, Y., Izumi, N., Nishikawa, S., Jakt, M.L., Tarui, H., and Nishikawa, S.I. (2011). Etv2/ER71 induces vascular mesoderm from Flk1+PDGFRα + primitive mesoderm. Blood *118*, 6975–6986. https://doi.org/10.1182/blood-2011-05-352658.

26. Koyano-Nakagawa, N., Kweon, J., Iacovino, M., Shi, X., Rasmussen, T.L., Borges, L., Zirbes, K.M., Li, T., Perlingeiro, R.C.R., Kyba, M., et al. (2012). Etv2 is expressed in the yolk sac hematopoietic and endothelial progenitors and regulates Lmo2 gene expression. Stem Cells *30*, 1611–1623. https://doi.org/10.1002/stem.1131.

27. Fleury, M., Eliades, A., Carlsson, P., Lacaud, G., and Kouskoff, V. (2015). FOXF1 inhibits hematopoietic lineage commitment during early mesoderm specification. Development *142*, 3307–3320. https://doi.org/10.1242/dev.124685.

28. Nahaboo, W., Eski, S.E., Despin-Guitard, E., Vermeersch, M., Versaevel, M., Saykali, B., Monteyne, D., Gabriele, S., Magin, T.M., Schwarz, N., et al. (2022). Keratin filaments mediate the expansion of extra-embryonic membranes in the post-gastrulation mouse embryo. EMBO J. *41*, e108747. https://doi.org/10.15252/embj.2021108747.

29. Scotti, M., Kherdjemil, Y., Roux, M., and Kmita, M. (2015). A Hoxa13:Cre mouse strain for conditional gene manipulation in developing limb, hindgut, and urogenital system. Genesis *53*, 366–376. https://doi.org/10.1002/dvg.22859.

30. Naiche, L.A., Arora, R., Kania, A., Lewandoski, M., and Papaioannou, V.E. (2011). Identity and fate of Tbx4-expressing cells reveal developmental cell fate decisions in the allantois, limb, and external genitalia. Dev. Dyn. *240*, 2290–2300. https://doi.org/10.1002/dvdy.22731.

31. Schemmer, J., Araúzo-Bravo, M.J., Haas, N., Schäfer, S., Weber, S.N., Becker, A., Eckert, D., Zimmer, A., Nettersheim, D., and Schorle, H. (2013). Transcription Factor TFAP2C Regulates Major Programs Required for Murine Fetal Germ Cell Maintenance and Haploinsufficiency Predisposes to Teratomas in Male Mice. PLoS One *8*, e71113. https://doi.org/10.1371/JOURNAL.PONE.0071113.

32. Saitou, M., Barton, S.C., and Surani, M.A. (2002). A molecular programme for the specification of germ cell fate in mice. Nature *418*, 293–300. https://doi.org/10.1038/nature00927.

33. Lugus, J.J., Park, C., Ma, Y.D., and Choi, K. (2009). Both primitive and definitive blood cells are derived from Flk-1 + mesoderm. Blood *113*, 563–566. https://doi.org/10.1182/blood-2008-06-162750.

34. Bisia, A.M., Costello, I., Xypolita, M.E., Harland, L.T.G., Kurbel, P.J., Bikoff, E.K., and Robertson, E.J. (2023). A degron-based approach to manipulate Eomes functions in the context of the developing mouse embryo. Proc. Natl. Acad. Sci. USA *120*, e2311946120. https://doi.org/10.1073/pnas.2311946120.

35. Yzaguirre, A.D., de Bruijn, M.F.T.R., and Speck, N.A. (2017). The role of Runx1 in embryonic blood cell formation. Adv. Exp. Med. Biol. *962*, 47–64. https://doi.org/10.1007/978-981-10-3233-2_4.

36. Liu, F., Li, D., Yu, Y.Y.L., Kang, I., Cha, M.J., Kim, J.Y., Park, C., Watson, D. K., Wang, T., and Choi, K. (2015). Induction of hematopoietic and endothelial cell program orchestrated by ETS transcription factor ER 71/ ETV 2. EMBO Rep. *16*, 654–669. https://doi.org/10.15252/embr.201439939.

37. Rasmussen, T.L., Kweon, J., Diekmann, M.A., Belema-Bedada, F., Song, Q., Bowlin, K., Shi, X., Ferdous, A., Li, T., Kyba, M., et al. (2011). ER71 directs mesodermal fate decisions during embryogenesis. Development *138*, 4801–4812. https://doi.org/10.1242/dev.070912.

38. Koyano-Nakagawa, N., and Garry, D.J. (2017). Etv2 as an essential regulator of mesodermal lineage development. Cardiovasc. Res. *113*, 1294–1306. https://doi.org/10.1093/cvr/cvx133.

39. Nowotschin, S., Costello, I., Piliszek, A., Kwon, G.S., Mao, C.A., Klein, W. H., Robertson, E.J., and Hadjantonakis, A.K. (2013). The T-box transcription factor Eomesodermin is essential for AVE induction in the mouse embryo. Genes Dev. *27*, 997–1002. https://doi.org/10.1101/gad.215152.113.

40. Loh, K.M., and Ang, L.T. (2024). Building human artery and vein endothelial cells from pluripotent stem cells, and enduring mysteries surrounding arteriovenous development. Semin. Cell Dev. Biol. *155*, 62–75. https://doi.org/10.1016/j.semcdb.2023.06.004.

41. Dann, E., Henderson, N.C., Teichmann, S.A., Morgan, M.D., and Marioni, J.C. (2021). Differential abundance testing on single-cell data using k-nearest neighbor graphs. Nat. Biotechnol. *2021*, 1–9. https://doi.org/10.1038/s41587-021-01033-z.

42. Senft, A.D., Bikoff, E.K., Robertson, E.J., and Costello, I. (2019). Genetic dissection of Nodal and Bmp signalling requirements during primordial germ cell development in mouse. Nat. Commun. *10*, 1089. https://doi.org/10.1038/s41467-019-09052-w.

43. Inman, K.E., and Downs, K.M. (2006). Brachyury is required for elongation and vasculogenesis in the murine allantois. Development *133*, 2947–2959. https://doi.org/10.1242/dev.02454.

44. Zhao, H., and Choi, K. (2017). A CRISPR screen identifies genes controlling Etv2 threshold expression in murine hemangiogenic fate commitment. Nat. Commun. *8*, 541. https://doi.org/10.1038/s41467-017-00667-5.

45. Ying, Q.L., Wray, J., Nichols, J., Batlle-Morera, L., Doble, B., Woodgett, J., Cohen, P., and Smith, A. (2008). The ground state of embryonic stem cell self-renewal. Nature *453*, 519–523. https://doi.org/10.1038/nature06968.

46. Nostro, M.C., Cheng, X., Keller, G.M., and Gadue, P. (2008). Wnt, Activin, and BMP Signaling Regulate Distinct Stages in the Developmental Pathway from Embryonic Stem Cells to Blood. Cell Stem Cell *2*, 60–71. https://doi.org/10.1016/j.stem.2007.10.011.

47. Barile, M., Imaz-Rosshandler, I., Inzani, I., Ghazanfar, S., Nichols, J., Marioni, J.C., Guibentif, C., and Göttgens, B. (2021). Coordinated changes in gene expression kinetics underlie both mouse and human erythroid maturation. Genome Biol. *22*, 197. https://doi.org/10.1186/S13059-021-02414-Y.

48. Lun, A.T.L., McCarthy, D.J., and Marioni, J.C. (2016). A step-by-step workflow for low-level analysis of single-cell RNA-seq data with Bioconductor. F1000Res *5*, 2122. https://doi.org/10.12688/f1000research.9501.2.

49. McCarthy, D.J., Campbell, K.R., Lun, A.T.L., and Wills, Q.F. (2017). Scater: Pre-processing, quality control, normalization and visualization of single-cell RNA-seq data in R. Bioinformatics *33*, 1179–1186. https://doi.org/10.1093/bioinformatics/btw777.

50. Bais, A.S., and Kostka, D. (2020). Scds: Computational annotation of doublets in single-cell RNA sequencing data. Bioinformatics *36*, 1150–1158. https://doi.org/10.1093/bioinformatics/btz698.

51. Haghverdi, L., Lun, A.T.L., Morgan, M.D., and Marioni, J.C. (2018). Batch effects in single-cell RNA-sequencing data are corrected by matching mutual nearest neighbors. Nat. Biotechnol. *36*, 421–427. https://doi.org/10.1038/nbt.4091.

52. Hao, Y., Stuart, T., Kowalski, M.H., Choudhary, S., Hoffman, P., Hartman, A., Srivastava, A., Molla, G., Madad, S., Fernandez-Granda, C., et al. (2024). Dictionary learning for integrative, multimodal and scalable single-cell analysis. Nat. Biotechnol. *42*, 293–304. https://doi.org/10.1038/s41587-023-01767-y.

53. Granja, J.M., Corces, M.R., Pierce, S.E., Bagdatli, S.T., Choudhry, H., Chang, H.Y., and Greenleaf, W.J. (2021). ArchR is a scalable software package for integrative single-cell chromatin accessibility analysis. Nat. Genet. *53*, 403–411. https://doi.org/10.1038/s41588-021-00790-6.

54. Argelaguet, R., Arnol, D., Bredikhin, D., Deloro, Y., Velten, B., Marioni, J.C., and Stegle, O. (2020). MOFA+: a probabilistic framework for comprehensive integration of structured single-cell data. Preprint at bioRxiv. https://doi.org/10.1101/837104.

55. Street, K., Risso, D., Fletcher, R.B., Das, D., Ngai, J., Yosef, N., Purdom, E., and Dudoit, S. (2018). Slingshot: cell lineage and pseudotime inference for single-cell transcriptomics. BMC Genomics *19*, 477. https://doi.org/10.1186/s12864-018-4772-0.

56. Van den Berge, K., Roux de Bézieux, H., Street, K., Saelens, W., Cannoodt, R., Saeys, Y., Dudoit, S., and Clement, L. (2020). Trajectory-based differential expression analysis for single-cell sequencing data. Nat. Commun. *11*, 1201. https://doi.org/10.1038/s41467-020-14766-3.

57. Chen, Y., Lun, A.T.L., and Smyth, G.K. (2016). From reads to genes to pathways: Differential expression analysis of RNA-Seq experiments using Rsubread and the edgeR quasi-likelihood pipeline. F1000Res *5*, 1438. https://doi.org/10.12688/F1000RESEARCH.8987.2.

CellPress
OPEN ACCESS

STAR★METHODS

KEY RESOURCES TABLE

REAGENT or RESOURCE	SOURCE	IDENTIFIER
Antibodies		
Pecam/CD-31 rat IgG antibody	BD Pharmingen	Cat# 550274; RRID: AB_393571
RFP rabbit IgG	Rockland antibodies	Cat# 600-401-379
Alexa Fluor 594 donkey anti-rabbit IgG	Invitrogen/Life Technology	Cat# A21207; RRID: AB_141637
Alexa Fluor 488 donkey anti-rat IgG	Invitrogen/Life Technology	Cat# A21208; RRID: AB_2535794
polyclonal HRP conjugated Anti-goat IgG	abcam	Cat# ab6741; RRID: AB_955424
polyclonal HRP conjugated Anti-rat IgG	Cell Signaling	Cat# 7077S; RRID: AB_10694715
Anti-T goat polyclonal	Santa Cruz	Cat# sc-17745; RRID: AB_2200243
Anti-EOMES rat monoclonal	Thermo Fisher	Cat# 14-4875-82; RRID: AB_11042577
Alexa Fluor™ 488 anti-EOMES rat monoclonal	Invitrogen	Cat# 53-4875-82; RRID: AB_10854265
Pe/Cy7 anti-mouse FLK-1	BioLegend	Cat# 136414; RRID: AB_2561606
BV605 rat anti-mouse PDGFRA	BD Biosciences	Cat# 740380; RRID: AB_2740111
APC anti-mouse DLK1	R&D Systems	Cat# FAB8634A; RRID: AB_2890004
Pe/Cy7 anti-mouse CDH5	Thermo Fisher Scientific	Cat# 25-1441-82; RRID: AB_2573402
APC anti-mouse CD41	Thermo Fisher Scientific	Cat# 17-0411-82; RRID: AB_1603237
Chemicals, peptides, and recombinant proteins		
Vectashield mounting medium with DAPI	Vector Laboratories	Cat# H-1200
DAPI	BD Bioscience	Cat# 564907
eBioscience™ Fixable Viability Dye eFluor™ 450	Invitrogen	Cat# 65-0863-14
PD0325901	Synthesized by the MRC Protein Phosphorylation Unit, Division of Signal Transduction Therapy at the University of Dundee, UK	N/A
CHIR99021	Synthesized by the MRC Protein Phosphorylation Unit, Division of Signal Transduction Therapy at the University of Dundee, UK	N/A
LIF	Millipore	Cat# ESG1107
Recombinant human VEGF	R&D Systems	Cat# 293-VE
Recombinant human BMP4	R&D Systems	Cat# 314-BP
Recombinant human/mouse/rat Activin A	R&D Systems	Cat# 338-AC
dTAG-13	Tocris / Bio-Techne	Cat# 6605
β-mercaptoethanol	Sigma-Aldrich	Standard reagent
TrypLE Express	Thermo Fisher Scientific	Cat# 12605010
Neurobasal media	Gibco	Cat# 21103049
DMEM/F12	Gibco	Cat# 11320033
B27	Gibco	Cat# 17504044
BSA	Gibco	Cat# 15260037
Pen/Strep	Thermo Fisher Scientific	Cat# 15140122
L-glutamine	Thermo Fisher Scientific	Cat# 25030024
DMSO	Merck	D2650-100ML
Critical commercial assays		
DC™ Protein Assay Kit I	Biorad	Cat# 5000111
Laemmli buffer	Biorad	Cat# 1610747
EveryBlot blocking buffer	Biorad	Cat# 12010020
ECL™ prime Western Blotting Detection Reagent	Sigma-Aldrich	Cat# GERPN2236

(*Continued on next page*)

Continued		
REAGENT or RESOURCE	SOURCE	IDENTIFIER
eBioscience™ Fixable Viability Dye eFluor™ 450	Invitrogen	Cat# 65-0863-14
eBioscience™ Foxp3/ Transcription Factor Staining Buffer Set	Invitrogen	Cat# 00-5523-00
Deposited data		
In vivo Eomes chimera 10x scRNA-seq	This paper	GEO: GSE274166
In vitro differentiation 10x genomics RNA+ATAC	This paper	GEO: GSE274167
In vitro chromatin accessibility profiles	This paper	https://genome.ucsc.edu/s/Barttheeuwes/Theeuwes_Harland_vitro_multiome
Code for all bio-informatics analysis	This paper	https://github.com/BartTheeuwes/Eomes_ExEM
Experimental models: Cell lines		
tdTomato-wildtype mESCs	Pijuan-Sala et al.[17]	N/A
T-KO tdTomato mESCs	Guibentif et al.[14]	N/A
Eo-KO tdTomato mESCs	This paper	N/A
Eo/T-KO tdTomato mESCs	This paper	N/A
E14 Runx-Venus $Eo^{deg/deg}$ (clone G9)	Bisia et al.[34]	N/A
E14 Runx-Venus $Eo^{deg/deg}$ T-null (clones G9/A11, G9/F9)	This paper	N/A
Experimental models: Organisms/strains		
C57BL/6 wildtype mice	Charles River	C57BL/6J
Oligonucleotides		
Alt-R® CRISPR-Cas9 tracrRNA	IDT DNA	N/A
Alt-R CRISPR-Cas9 crRNA: Mm.Cas9.T.1.AD [sequence(PAM)] [GGTGGTCCACTCGGTACTGC(AGG)]	IDT DNA	N/A
ssODN: t*c*tcctccaggcccactcgcagttcgcgttcggtggggtct cccttctcgctgcccgcctgcagctcgctctccacggcgctgagcaggt ggtccactcgactagttcatcagtactgcagactcttccctgcgctctctg tgcccggcgagctcatcctcccgccaccctctccacctt*c*c	IDT DNA	N/A
Primer AB_289: CTCCGCAGAGTGACCCTTTT	IDT DNA	N/A
Primer AB_290: TACCTGCCGTTCTTGGTCAC	IDT DNA	N/A
Alt-R CRISPR-Cas9 crRNA: gRNA#2 [sequence(PAM)] [GGTTTGCAGCAGGCGATTTG(TGG)]	Harland et al.[10]	N/A
Alt-R CRISPR-Cas9 crRNA: gRNA#3 [sequence(PAM)] [AGAGGCATCCCGGCACCCTG(AGG)]	Harland et al.[10]	N/A
ssODN#2 (SphI): C*C*CTCTTAACCTCCCTCCCCATG CCCTAAATAAACTCTATTCTATACTATTCCATCTTGT GGCTGGTCCCTCAGGGCATGCGCCTGCTGCAAA CCCAGGAGCCAGCGGGTCACGTAGATCTGCCCT CAAGGGTTCATTCCCAAATTTCCATC*T*C	Harland et al.[10]	N/A
ssODN#3 (SpeI): C*C*CTCTTAACCTCCCTCCCCATG CCCTAAATAAACTCTATTCTATACTATTCCATCTTGT GGCTGGTCCCTCAGGACTAGTGCCTGCTGCAAAC CCAGGAGCCAGCGGGTCACGTAGATCTGCCCTCA AGGGTTCATTCCCAAATTTCCATC*T*C	Harland et al.[10]	N/A
Software and algorithms		
cellranger-arc	10x Genomics Inc.	N/A
R	N/A	https://www.R-project.org/
Scran	Bioconductor	https://bioconductor.org/packages/release/bioc/html/scran.html
Scuttle	Bioconductor	https://bioconductor.org/packages/release/bioc/html/scuttle.html

(*Continued on next page*)

Continued

REAGENT or RESOURCE	SOURCE	IDENTIFIER
Scds	Bioconductor	https://www.bioconductor.org/packages/release/bioc/html/scds.html
Batchelor	Bioconductor	https://bioconductor.org/packages/release/bioc/html/batchelor.html
BiocNeighbors	Bioconductor	https://www.bioconductor.org/packages/release/bioc/html/BiocNeighbors.html
irlba	Cran	https://cran.r-project.org/web/packages/irlba/index.html
Seurat	Cran	https://cloud.r-project.org/web/packages/Seurat/
Scater	Bioconductor	https://www.bioconductor.org/packages/release/bioc/html/scater.html
ArchR	GitHub	https://github.com/GreenleafLab/ArchR
motifmatchr	Bioconductor	https://bioconductor.org/packages/release/bioc/html/motifmatchr.html
MOFA+	Bioconductor	https://www.bioconductor.org/packages/release/bioc/html/MOFA2.html
uwot	Cran	https://cran.r-project.org/web/packages/uwot/
edgeR	Bioconductor	https://bioconductor.org/packages/release/bioc/html/edgeR.html
Slingshot	Bioconductor	https://www.bioconductor.org/packages/release/bioc/html/slingshot.html
TradeSeq	Bioconductor	https://www.bioconductor.org/packages/release/bioc/html/tradeSeq.html
CellRanger	10x Genomics Inc.	N/A
MouseGastrulationData	Bioconductor	https://bioconductor.org/packages/release/data/experiment/html/MouseGastrulationData.html
MiloR	Bioconductor	https://www.bioconductor.org/packages/release/bioc/html/miloR.html
FlowJo (10.10.0)	BD Biosciences	https://www.flowjo.com/
Other		
10x Genomics single-cell 3' RNA-seq (v3)	10x Genomics Inc.	N/A
10x Genomics Epi Multiome ATAC + Gene Expression	10x Genomics Inc.	N/A

EXPERIMENTAL MODELS AND STUDY PARTICIPANTS

ESC genetic modification using CRISPR-Cas9

Generating TdTomato+ Eo-KO and TdTomato+ Eo/T dKO for chimera-seq

For the generation of TdTomato+ Eo-KO and TdTomato+ Eo/T dKO, two custom crRNAs were used to generate an Eomes loss-of-function allele[8] as described previously[10] using TdTomato+[17] (male) and tdTomato+ T-null ESCs lines[14] (male), respectively. Briefly, ssODNs were designed that contained a 5′ homology arm upstream of the Eomes intron 1 DNA double-strand break site, followed by insertion of a new EcoRV, Sph1 or Spe1 restriction site and a 3′ homology arm located downstream of the Eomes intron 5 DNA break site (key resources table). 10 μl of 2×10^4 ESCs in Buffer R were electroporated with 1 μl of a 1:1 mix of the RNPs and 2 μl of the ssODN. Low-density plating was performed after 72 hours, and after 7–10 days clones were picked and screened using a three-primer PCR strategy that simultaneously amplified the WT allele and the null allele (key resources table). Genotypes of clones were verified using PCR, followed by restriction enzyme digests and Western blotting.

Eo-deg T-KO for EB differentiations

Feeder-free E14 Runx-Venus Eomes$^{deg/deg}$ ESCs[34] were targeted with a Cas9-mediated strategy like that described above, using ssODN template and crRNA guides designed to generate a T-null modification (key resources table). Genotypes of clones were verified using PCR, followed by restriction enzyme digests and Western blotting.

ESC maintenance

All TdTomato+ mouse ESC lines were cultured at 37C under 2i+LIF conditions[45] prior to blastocyst injection for chimera-seq experiments. Runx1^{Venus} Eomes$^{mCherry\text{-}degron}$ ESC lines and their derivatives were maintained in feeder free culture conditions in serum + LIF on gelatinized plates.[9]

In vitro hematovascular EB differentiation protocol

48-72 hours prior to induction of hematovascular differentiation ESC were washed with PBS and cultured in serum free ESC media containing 50% Neurobasal Media (Gibco, Cat #21103049), 50% DMEM/F12 (Gibco, Cat#11320033), supplemented with 0.5X of both N2 (Gibco, Cat #17502048) and B27 (Gibco, Cat #17504044), 1% Pen/Strep, 1% glutamine, 0.05% BSA (Gibco, Cat #15260037), 1 μM PD0325091, 3 μM CHIR99021 and 1000 U/ml LIF. At day 0 cells were dissociated using TrypLE Express (Thermo Fisher Scientific, Cat# 12605010) and seeded at a density of 1x10^5 cells/mL in serum-free differentiation (SF-D) media[46] and cultured on an orbital shaker at 70 rpm for ∼18 hours in the absence of growth factors to form EBs. At day 2, EBs were split 1:3 in SF-D media containing recombinant human (rh) VEGF (5 ng ml-1; R&D Systems), rhBMP4 (10 ng ml-1; R&D Systems) and Activin A (5 ng ml-1; R&D Systems) for 48 hr. At day 3, EBs were treated with either DMSO for WT controls or dTAG-13 for Eomes-KD (100 nM, Biotechne Tocris, Cat #6605). At day 4, EBs were split 1:2 in SF-D media containing rhVEGF (5 ng ml-1; R&D Systems), rhBMP4 (10 ng ml-1; R&D Systems) and Activin A (5 ng ml-1; R&D Systems) for 24 hours.

Chimera generation

tdTomato-ESC clones used for chimera generation included Eomes-KO (this study), T-KO,[14] Eomes/T-DKO cells (this study) and wild-type controls.[17] E3.5 blastocysts (male and female) were derived from wildtype C57BL/6 matings, injected with 4-6 ESC and transferred into 2.5 day pseudopregnant recipient females. This protocol allows the recovery of chimeric embryos with a 5-50% ESC contribution as previously described.[14,17,47] Chimeric embryos were harvested at E8.5, dissected and processed for either imaging or scRNA-seq. Embryos for scRNA-seq were dissociated using TrypLE Express dissociation reagent (Thermo Fisher Scientific) incubation for 7-10 min at 37°C under agitation followed by sorting of single-cell suspensions into tdTomato+ (KO) and tdTomato- (WT) samples using a BD Influx sorter with DAPI at 1 mg/ml (Sigma) and subsequent 10x Genomics single-cell 3' RNA-seq (v3). 4 separate Eomes-KO clones were used, and each replicate consists of multiple embryos pooled together from individual clones. Chimeric embryo pool efficiencies ranged from approximately 5 to 30%. All mice were bred and maintained in microisolator cages and provided continuously with sterile food, water, and bedding. All mice were kept in specified pathogen-free conditions. All Experiments were performed in accordance with EU guidelines for the care and use of laboratory animals and under the authority of appropriate UK governmental legislation. Use of animals in this project was approved by the Animal Welfare and Ethical Review Body for the University of Cambridge and University of Oxford, covered by relevant Home Office licences.

METHOD DETAILS

Western blot

EBs were lysed in RIPA (50 mM Tris pH8.0, 150 mM NaCl, 1% Igepal, 0.5% Na deoxycholate, 0.1% SDS). Protein quantification by Bradford was carried out using DC™ Protein Assay Kit I (BioRad) on a Jenway Genova DNA Life Science Analyzer. Samples were denatured at 98°C for 10 min in Laemmli buffer (BioRad) with 10% β-mercaptoethanol, run on a Mini-Protean® PAGE gel (BioRad) at 90V and transferred onto PVDF membrane for 75 min at 90V. The membrane was rinsed with dH2O, washed in 0.1% TBST (Tris-buffered saline-Tween20) for 10 min, blocked with EveryBlot blocking buffer (BioRad) for 10 min at room temperature incubated with primary antibody on a shaking platform overnight at 4°C, washed with TBST, incubated in secondary antibody, and washed with TBST. ECL prime (Sigma-Aldrich) was added per manufacturer's instructions and exposed to X-ray film. When required, the membrane was stripped with stripping buffer (2.9 g glycine, 20 ml SDS in 2L H2O, pH 2.2), blocked, washed, and exposed to antibody as above. Antibodies used are listed in the key resources table.

Flow cytometry

Live flow cytometry

For live flow cytometry, EBs were washed with PBS, dissociated with TrypLE and neutralised with FACS buffer (PBS with 1% Pen/Strep, 2% FCS (fetal calf serum)). Cells were resuspended in FACS buffer containing fluorophore-conjugated antibodies and stained on ice for 30 min. Cells were washed and resuspended in FACS buffer with 1:5,000 DAPI (BD Biosciences) for 15 min on ice, washed and resuspended again in FACS buffer. Antibodies used are listed in the key resources table.

Intracellular flow cytometry

For intracellular flow cytometry, EBs were washed with PBS, dissociated with TrypLE and neutralized with FACS buffer (PBS with 1% Pen/Strep, 2% FCS (fetal calf serum)). Next, cells were stained with an eBioscience™ Fixable Viability Dye eFluor™ 450 (Invitrogen, Cat# 65-0863-14) in a 96-well plate for approximately 30 min, washed with PBS and subsequently fixed and permeabilized using the eBioscience™ Foxp3/Transcription Factor Staining Buffer Set (Invitrogen, Cat# 00-5523-00) following manufacturer instructions. Cells were stained with an anti-Eomes AF88 antibody (Invitrogen, Cat# 53-4875-82) at a concentration of 1:400 for 30 min at room temperature, washed and resuspended again in FACS buffer for flow cytometric analyses.

Collecting EB samples for 10x multiomics and flow cytometry

In two separate experiments (EB differentiations performed weeks apart) live and intracellular flow cytometry were performed on day 3, 3.5, 4, 4.5 and 5 WT and Eomes-KD EB samples to track the emergence of HE and blood progenitor populations by assessing the expression of key markers: CD41 and Runx1 (blood progenitor markers), CDH5 (endothelial marker), and KDR/PDGFRA (Figure S3). In the same experiments we also performed the 10x Genomics multiomic workflow (Figures 1 and 2), on day 3, 3.5, 4, 4.5 and 5 EB samples. Notably, the start time of the differentiation setup was staggered, which allowed the collection and dissociation of day 3, 3.5, 4, 4.5, and 5 EBs from both DMSO and dTAG treated conditions on the same collection day. After dissociating the EBs into single cells using TrypLE each sample was split: half of the cells were taken for 10x Genomics multiomic workflow and the other half for live and intracellular flow cytometry. Intracellular flow cytometry confirmed the depletion of Eomes protein expression levels after dTAG addition to the cultures in these experiments (Figure S3).

Nuclear extraction and 10x genomics multiome library preparation

Cell suspensions in 2 mL eppendorf tubes were centrifuged at 300 xg for 5 min, resuspended in 1 mL of PBS supplemented with 0.04% BSA then filtered using a 40 μM flowmi cell strainer. After centrifuging again, the supernatant was removed and the cell pellet resuspended in 100 μl of ice-cold nuclear extraction (NE) buffer (10 mM Tris pH 7.5, 10 mM NaCl, 3 mM MgCl2, 1% BSA, 0.1% Tween, 1 mM DTT, 1 U/μl RNaseIn (Promega), 0.1% NP40, 0.01% Digitonin) and incubated on ice for 4 min. 1mL of wash buffer (identical to NE buffer but lacking NP40 and digitonin) was added and nuclei were centrifuged at 500 xg for 5 min at 4°C. Nuclei washed twice by resuspension in 1 mL of wash buffer and centifugation at 500 xg for 5 min at 4°C. Nuclei were resuspended in 50 μl of diluted nuclei buffer (10x Genomics) and 1 μl was used to assess quality using a microscope and count nuclei using a Countess II instrument. >99% of nuclei stained positive for trypan blue and the nuclei were found to have the expected morphology. Nuclei were diluted such that a maximum of 16,000 were taken forward for 10x Genomics multiome library preparation. Libraries were prepared using the 10x Genomics Multiome reagents and sequenced on a Novaseq 6000 instrument (Illumina) using the recommended read-lengths.

In vitro RNA+ATAC Multiome pre-processing

Mapping sequencing data

10x Genomics multiome raw base call BCL were first demultiplexed using cellranger-arc mkfastq (Cell Ranger ARC version 2.0.1) to get separate FASTQ files for the GEX (RNA) and ATAC libraries. Next, FASTQ files were mapped to the mm10 reference genome supplied by 10x Genomics (arc-mm10-2020-A-2.0.0) using cellranger-arc count. All downstream processing and analysis were performed in R (v4.2.2). Analysis started with the pre-processing of the RNA data, followed by pre-processing the ATAC and analysis of the combined modalities.

scRNA-seq pre-processing

From the scRNA-seq, high quality cells were retained using the following thresholds: $\log_{10}$(number of reads) > 3 & < 5, number of genes > 2.5e3 & < 12e3, percentage mitochondrial RNA < 25%, percentage ribosomal RNA < 30%. Count normalization was performed by first calculating size factors using the computeSumFactors function from scran[48] (Version 1.26.0), where cells were pre-clustered using scran's quickCluster with method=igraph, minimum and maximum sizes of 100 and 3000 cells per cluster, respectively, followed by logNormCounts from scuttle[49] (Version 1.8.0). Doublet calling was performed with the scds[50] (Version 1.14.0) package using the hybrid approach and doublet score > 1.25 as threshold. A total of 35,125 cells passed RNA QC.

scRNA label transfer from the scRNA-seq atlas of gastrulation and early organogenesis

Label transfer for cell-type assignment was conducted by mapping to the E6.5 – E8.5 stages of the gastrulation atlas, with additional cell type annotation from an updated extended gastrulation atlas.[17,19] First, the reference atlas was subset to a maximum of 15,000 cells per embryonic stage, including all cells for the 'mixed-gastrulation' stage. Mapping was performed for each sample separately using the batchelor package[51] (Version 1.14.0), with each sample referred to as the 'query'. For mapping to the reference atlas, non-informative and batch-effect genes were excluded, including those with names starting or ending with Rik, Mt, Rps, Rpl, or Gm, as well as haemoglobin genes, imprinted genes Grb10 and Nnat. Subsequently, the top 2,500 genes with the highest variability in expression across atlas cell types were selected for integration. Joint normalization was performed using multiBatchNorm with batch being either 'query' or 'atlas', followed by multiBatchPCA with the same batch variable and d = 50. Batch correction was executed in multiple rounds using ReducedMNN. Initially, the atlas was corrected within each embryonic stage, ordering samples from largest to smallest. This was followed by correcting the atlas between embryonic stages, ordered from latest to earliest. Lastly, batch correction was applied between the reference atlas and the query. Nearest neighbors (NN) of each query cell in the reference atlas were identified using queryKNN from the BiocNeighbors package (Version 1.16.0) with k=25. Cell-type label transfer was then performed by determining the mode of the cell types of the NN, with ties resolved by assigning the cell type of the reference atlas cell closest to the query.

scRNA dimensionality reduction

Dimensionality reduction was performed on lognormalized counts by first calculating the top variable genes using Seurat's[52] (Version 4.3.0) FindVariableFeatures with nfeatures = 1500 after excluding the same non-informative and batch-effect genes as described above. Next, the number of reads and genes, as well as the percentage of mitochondrial and ribosomal reads were regressed out for the lognormalized counts. PCA was then performed using scater's[49] (Version 1.26.0) runPCA with ncomponents = 15.

scATAC pre-processing

Full scATAC-seq analysis was performed using the ArchR package[53] (Version 1.0.1). Arrow files were created from CellRangers fragment files using createArrowFiles using initial, relaxed, quality threshold for the transcription start site enrichment (min/max TSS) and number of frags (min/max Frags): minTSS = 2.5, minFrags = 1e3, and maxFrags = 1e6, while excluding chromosomes 'chrM' and 'chrY', followed by creation of the ArchR object using the ArchRProject function for the arrow files from all samples. After inspecting quality, cells were retained using the following, more stringent, thresholds: minTSS = 9, maxTSS = 35, and minFrags = 3.5e3, as well as a maximum proportion of reads in blacklisted genomic regions of 0.05. Cells detected as doublets in the RNA were excluded from downstream analysis, while cells that were only detected in the ATAC but not in the RNA, while passing ATAC QC thresholds, were kept. A total of 35,864 cells passed ATAC QC.

scATAC peak detection and dimensionality reduction

The mouse genome was split into 500 bp bins to create a count matrix with accessibility per bin for each cell using ArchR's addTileMatrix. Next, Latent Semantic Indexing (LSI) dimensionality reduction was performed on the TileMatrix using addIterativeLSI with iterations = 4, varFeatures = 2e4, dimsToUse = 1:30, and clusterParams' resolution = c(0.4, 1, 2) and maxClusters = NULL. The LSI was used for clustering data using addClusters with resolution = 0.5. Next, insertion coverage files were created for pseudobulked replicates of each TileMatrix LSI cluster and Eomes condition (KD or WT) combination using addGroupCoverages with groupBy = the cluster & condition combination, minCells = 50, maxCells = 5000. These insertion coverage files were used for Macs2 peak calling using the function addReproduciblePeakSet with groupBy = the cluster & condition combination, cutOff = 1e-3, and extendSummits = 300, for peaks with a width of 600 bp. A count matrix for the accessibility of those peaks per cell was created using addFeatureMatrix with the Granges object of the peaks as features, and ceiling = 4. LSI dimensionality reduction was performed on the PeakMatrix in the same way as described above for the TileMatrix, but with varFeatures = 2.5e4, dimsToUse = 1:20.

In-silico ChIP of the in vivo multiome atlas of murine embryology

In order to predict TF occupancy at each of the identified peaks from the *in vitro* 10x Genomics multiome data, we implemented the *in silico* ChIP method on the *in vivo* multiome gastrulation atlas as previously described.[18] Briefly, for all cells that pass quality thresholds in both the RNA and ATAC modality of the *in vivo* data, we first identified high resolution clusters needed to correlate TF expression with peak accessibility. Transcriptomics counts were lognormalised using Scuttle's logNormCounts, followed by identification of the top 5,000 highly variable genes, ordered by FDR, using Scran's modelGeneVar function with samples as block. Next, PCA was performed using runPCA with ncomponents = 40 on all HVGs. High resolution clustering was then performed using seurat's functions FindNeighbors across all PC dimensions and FindClusters with a resolution of 20 in order to identify 222 clusters. These clusters were used to pseudobulk the count matrices for the RNA expression and the ATAC accessibility of the *in vitro* peak set using Scuttle's aggregateAcrossCells. Pseudobulk matrices were normalized by dividing counts by total counts per cluster, multiplying by 1e6 and adding a pseudocount of 1, followed by taking the natural log. Next, motif detection in the *in vitro* peaks was performed using the cisbp dataset of the chromVARmotifs package (Version 0.2.0). To make motif names consistent with gene names, the Tcfap family was renamed to tfap. Motif annotation was performed using motifmatchr's (Version 1.20.0) matchMotifs function with out = 'scores', p.cutoff = 5e-5, and w = 7. Next, for every TF, we used psych's (Version 2.2.9) corr.test function to calculate the pearson correlation between the normalized counts of its expression with the normalized counts of the accessibility of peaks containing its respective motif. The *in silico* binding score for each TF to a peak was calculated by multiplying this correlation with the minmax normalized product of the TF motif score of the peak and the maximum normalized accessibility of the peak across all clusters. An *in silico* binding score of > 0.2 was set as a threshold for a peak to be predicted as 'bound' by a specific TF. The *in silico* ChIP motif annotation is used for any motif analysis throughout the paper.

scATAC ChromVAR

To calculate TF activity on a per-cell basis, we employ ArchR's ChromVAR implementation using the addDeviationsMatrix function, with the matches being the peak annotation for motifs with a predicted binding score of >0.2. TFs with fewer than 40 peaks predicted to be bound were excluded from downstream analysis.

scRNA & scATAC integration

For integration of the scRNA and scATAC modalities, we used the PCA and LSI calculated from the RNA and ATAC, respectively, scaled each component to achieve comparable ranges for both PCA and LSI, and performed MOFA+[54] (Version 1.8.0) using default settings on the subset of cells that passed QC for both modalities. This resulted in a total of 19 MOFA factors that were used for downstream analysis. UMAP was calculated from MOFA factors using uwot's (Version 0.1.14) umap function with n_neighbors = 25 and min_dist = 0.15. Clustering was performed on MOFA factors using Seurat's FindNeighbors with k.param = 35, followed by FindClusters with resolution = 1.5 in order to identify a total of 18 clusters.

Cluster marker genes were identified using Seurat's FindAllMarkers with min.pct = 0.5, logfc.threshold = 0.5, and only.pos = TRUE. Marker peaks were identified using ArchR's getMarkerFeatures, followed by motif enrichment using peakAnnoEnrichment with cutOff = "FDR <= 0.1 & Log_2FC >= 0.5" and plotting data was retrieved using plotEnrichHeatmap with returnMatrix = TRUE. Clusters with high overlap of transcriptomic markers and motif enrichment in chromatin accessibility markers were grouped together into 11 cell types. Cells annotated as PGCs via label transfer got their separate annotation. Annotation of these cell types is described in the following section ("Wild-type analysis: Cell type specific markers").

In vitro RNA+ATAC Multiome wild-type analysis

Cell type specific markers

The annotation of the final cell types was based on a combination of marker identification at the transcriptomic and chromatin accessibility level, as well as the proportion of each *in vivo* cell type that the *in vitro* cell types mapped to. Marker genes for the 12 cell types in the WT EBs were identified using Seurat's FindAllMarkers with min.pct = 0.1, logfc.threshold = 0.5, and only.pos = TRUE. ChromVAR marker motifs were identified using ArchR's getMarkerFeatures with useSeqnames="z", with Figure 1D showing $-\log_{10}$(FDR) displayed as the size and chromVAR Z-scores minmax normalized per motif. Marker peaks were identified using ArchR's getMarkerFeatures, followed by motif enrichment using peakAnnoEnrichment with cutOff = "FDR <= 0.1 & Log_2FC >= 0.5" and plotting data was retrieved using plotEnrichHeatmap with returnMatrix = TRUE. Atlas cell type label transfer proportions were plotted for the WT samples for each final cell type. Projection of in vitro cells on the Atlas UMAP was performed by taking the UMAP coordinates of their nearest neighbor in the *in vivo* atlas.[17] Finally, the identified markers in both molecular layers were extensively cross-referenced with the literature, their patterns were matched with published atlases of gastrulation, and *in vivo* label transfer proportions were considered in order to acquire the final cell type label as used throughout the manuscript. Bigwigs for the 12 cell types were generated using ArchR's getGroupBW with default parameters.

Genome wide correlation of accessibility with in vivo cell types

To compare the chromatin accessibility profiles of the cell types identified *in vitro* with their closest *in vivo* counterparts from Argelaguet et al.,[18] we correlated peak accessibility levels between the two datasets. Atlas peaks were used to generate a new peak matrix of the *in vitro* data using ArchR's addFeatureMatrix with ceiling = 4. Next, we pseudobulked peak matrices by cell types using scuttle's aggregateAcrossCells function for both the *in vitro* cell types and the relevant cell types from the Argelaguet et al.[18] *in vivo* atlas using only WT samples. Pre-calculated marker peaks for the *in vivo* cell types were used to perform correlation between *in vitro* and *in vivo* cell types using the cor function with method = 'pearson'.

Trajectory inference and differential testing

Trajectory inference was performed using the slingshot package[55] (Version 2.6.0). Briefly, the data was limited to the cell types of Primitive Streak, Early Mes #1 and #2, Posterior Mesoderm, Allantois Precursor, HE Precursor, and HE. Slingshot trajectories and pseudo-times were calculated using the slingshot function, with cell types as clusterLabels, the 'Primitive Streak' as start.clus, and the first 10 dimensions of the MOFA factors as reducedDim. Cells were assigned to either the allantois or YS trajectory using the.assignCells function of the tradeSeq package[56] (Version 1.12.0), with the slingshot weights as input. Differential testing was restricted to WT cells within pseudo-time 2 and 10, and to the top 1e4 highly variable genes. Differential testing along pseudotime was performed by first running Tradeseq's fitGAM, with the described cells and slingshot pseudo-time, the hvgs as genes, nknots = 8, and with the multiome replicates used as the design matrix. Next, differential genes were detected by using patternTest and diffEndTest, both with l2fc = 1, and genes with an FDR < 0.05 were labelled as significant. For the differential chromatin accessibility, fitGAM was run using the same parameters, with the exception that the count matrix was replaced by the ATAC count matrix and the hvgs were the top 42,261 most variable peaks. patternTest and diffEndTest were used with l2fc = 0.25 and peaks with an FDR < 0.05 were labelled as significant. Smoothed peak accessibility was acquired via TradeSeq's predictSmooth function followed by min-max normalization per peak and clustered using kmeans clustering with centers = 10. Motif enrichment for peak clusters was performed using ArchR's.computeEnrichment.

In vitro RNA+ATAC Multiome Eomes knock-down analysis

Differential abundance testing

Differential abundance testing was performed at a single-cell level after excluding the day 3 WT cells. Briefly, for each timepoint we determined the global expected KD ratio (KD cells/total cells). Next, for each cell, we identified the top 100 nearest neighbours using BiocNeighbors' findKNN function using the MOFA factors. For each cell we then calculate the per cell expected KD ratio by taking the sum of global expected KD ratios normalized for the number of neighbours belonging to each timepoint and dividing by 100. P-values were calculated by performing a two-sided binomial test with a confidence level of 0.95, testing if the observed KD ratio is different from the per cell expected KD ratio, followed by FDR multiple testing comparison. Levels of differential abundance were defined as the per cell observed KD ratio divided by the per cell expected KD ratio, as displayed in Figure 2C.

Differential genes and accessibility in early cell types

Differential expression and accessibility testing between KD and WT cells was performed using edgeR[57] (Version 3.40.0) on pseudobulk replicates. Briefly, for each cell type – genotype condition we generated 5 pseudobulk replicates by randomly sampling 40% of cells for each replicate. Genes were filtered by setting minimum thresholds for expression levels and cellular detection rates and a set of non-informative genes were removed before running edgeR identify DEGs between Eomes KD and WT. Significance was determined as an absolute logFC > 0.5 and FDR < 0.05. Differentially accessible regions were determined in the same way, using the peak count matrix rather than the RNA expression count matrix.

Endothelium detailed analysis

The endothelium sub-population was further analyzed to identify differences between Eomes-KD and WT samples at higher resolution. An endothelium specific UMAP was generated with Scater's runUMAP function with n_neighbors = 15 and min_dist = 0.3 using the MOFA factors. Clustering was performed on MOFA factors using Seurat's FindNeighbors with k.param = 20, followed by FindClusters with resolution = 0.4. Endothelial related gene expression patterns per cluster and condition were visualized using Seurat's DotPlot function.

Eomes-KD vs WT analysis along allantois trajectory

To perform differential expression/accessibility analysis between the Eomes-KD and WT samples along the allantois trajectory, we used the slingshot trajectory inference from 'Trajectory inference and differential testing' while excluding the day 3 WT samples. Cells were assigned to either the allantois or YS trajectory using the.assignCells function of the tradeSeq package, with the slingshot weights as input. Differential testing was restricted to cells assigned to the allantois trajectory within pseudo-time 2 and 10. Differential gene expression testing along pseudo-time was performed by first running Tradeseq's fitGAM, with the described cells and slingshot pseudo-time, nknots = 8, the genes parameter was set to those genes that are expressed in at least 15% of Eomes-KD or WT cells, and with the multiome replicates used as the design matrix. Next, differential genes were detected by using conditionTest with l2fc = 0.5 and FDR correction was performed to correct for multiple testing. Genes with an FDR < 0.05 were labelled as significant. For the differential chromatin accessibility, fitGAM was performed using the same parameters, with the exception that the count matrix was replaced by the ATAC count matrix and the hvgs were the top 43255 most variable peaks. conditionTest was performed with default parameters, followed by FDR correction. Peaks with an FDR < 0.05 were labelled as significant. Smoothed peak accessibility was acquired via TradeSeq's predictSmooth function followed by min-max normalization per peak, and clustering using kmeans clustering with centers = 10. T and Eomes ChIP-seq data was downloaded from GSE128466.[13] Overlaps between ChIP-seq peak and accessibility peak were determined using Granges' findOverlaps function with minoverlap = 100.

Chimera imaging

Embryos were washed in PBS and fixed in 4% paraformaldehyde overnight at 4°C. After three washes in PBS containing 0.1% Triton X-100 (PBS-T), samples were permeabilised in PBS containing 0.5% Triton X-100 at RT for 20 mins, followed by further washes in PBS-T. Embryos were blocked in 5% donkey serum, with 0.2% BSA in PBS-T at RT for 2 hours then incubated overnight with primary antibodies in blocking buffer at 4°C. After multiple washes in PBS-T, embryos were incubated with fluorophore-conjugated secondary antibodies in blocking buffer for 2 hours at RT, followed by further washes in PBS-T, including a PBS-T/DAPI wash. Embryos were mounted in Vectashield with DAPI on coverslip dishes and imaged on an Olympus FluoView FV1000 microscope and image data were processed using ImageJ software. Antibodies are listed in key resources table.

Chimera-seq analysis

Mapping sequencing data and Pre-processing

Eomes 10x Genomic scRNA-seq FASTQ files were mapped to the mm10 reference genome and counted with the GRCm38.p5 annotation including the Tomato-Td gene using CellRanger count (v6.0.1) with chemistry=SC3Pv3. E8.5 T chimera data for reanalysis was downloaded using the package MouseGastrulationData (Version 1.12.0) with the function TChimeraData with type = 'processed' and samples = c(1:2, 5:10). Eomes and T chimera scRNA-seq analysis was performed with the same parameters unless indicated. Chimera scRNA-seq pre-processing was performed in the same way as described above for the scRNA of the multiome assay, with a few exceptions. The thresholds were adjusted to $\log_{10}$(number of reads) > 3.5 & < 5, number of genes > 1.5e3 & < 1e4, percentage mitochondrial RNA < 5%, percentage ribosomal RNA < 35%. Doublet detection was performed as described, with a threshold of > 0.5 set as a doublet call. For the newly generated Eomes chimera-seq, a total of 22,600 WT and 9768 Eomes-KO cells were retained for downstream analysis. For the re-analysis of the E8.5 T chimera-seq, a total of 13,771 WT and 14,460 T-KO were retained.

Mapping and refined cell type annotation

Chimera scRNA-seq mapping to the reference atlas was performed in the same way as described above for the scRNA of the multiome assay in the 'scRNA label transfer from the scRNA-seq atlas of gastrulation and early organogenesis' section, with a few exceptions. Query cells were only mapped to atlas cells spanning stages E7.5 to E8.5. Additionally, genes starting or ending with Rik, Mt, Rps, Rpl, or Gm, haemoglobin genes, imprinted genes, sex related genes Xist, Tsix, and y-chromosomal genes, and td-Tomato itself were excluded for mapping and multiBatchPCA was performed on the top 4,000 most variable genes in the atlas with d = 40. After batch correction, the label of the 15 NN of the reference were used for label transfer. In order to increase the resolution in annotation for the cell types of interest, relevant labels from the original and extended gastrulation atlas were combined and used for downstream analysis.[17,19]

Milo differential abundance testing

Differential abundance testing was performed for each embryonic stage separately using the MiloR package[41] (Version 1.6.0). Cells with an annotation of "Visceral_endoderm", "ExE_endoderm", "ExE_ectoderm", or "Parietal_endoderm" were removed from the analysis. Dimensionality reduction was performed by first selecting the top 4000 highly variable genes of the WT samples, identified by Seurat's FindVariableFeatures, excluding genes starting or ending with Rik, Mt, Rps, Rpl, or Gm, haemoglobin genes, imprinted genes, sex related genes Xist, Tsix, and y-chromosomal genes, and td-Tomato itself. Next, the number of reads and genes, as well as the percentage of mitochondrial and ribosomal reads were regressed out for the lognormalized counts. Principle component analysis was performed using multiBatchPCA with the genotype set as batch variable and d = 40, followed by batch correction using reducedMNN. Next, Milo was performed on batch corrected PCs by first running buildGraph with k = 20 for Eomes and k = 40 for T chimeras, followed by makeNhoods with the same values for k and prop=0.1. Cells per neighbourhood were counted using countCells and differential abundance testing was performed using testNhoods with design = ~ embryo pool + genotype, where each embryo pool contains one sample for both the KO and the WT condition that were collected as chimeric embryos and separated by the presence or absence of tdTomato expression by fluorescence-activated cell sorting.

Differential gene expression testing

Differential gene expression testing was performed by first aggregating cells by their sample, genotype, and cell type annotation. Then, using scran's pseudoBulkSpecific with design = ~ embryo pool + genotype, we identified genes that were differentially expressed between the KO and WT cells in a cell type specific way, with p-values being calculated by comparing gene specific logFC to the average logFC across all cell types. Significance was determined as an absolute logFC > 0.5 and FDR < 0.05. For comparison between T and Eomes DEGs, genes that were excluded from one of the cell types in one of the KOs were set as a logFC of 0.

QUANTIFICATION AND STATISTICAL ANALYSIS

Statistical details for each analysis, including how significance was defined, are described in the relevant section of the "method details" in the STAR Methods.

Developmental Cell, Volume *60*

Supplemental information

Eomes directs the formation of spatially and functionally diverse extraembryonic hematovascular tissues

Bart Theeuwes, Luke T.G. Harland, Alexandra M. Bisia, Ita Costello, Mai-Linh N. Ton, Tim Lohoff, Stephen J. Clark, Ricard Argelaguet, Nicola K. Wilson, Wolf Reik, Elizabeth K. Bikoff, Elizabeth J. Robertson, and Berthold Göttgens

SUPP. FIGURE 1

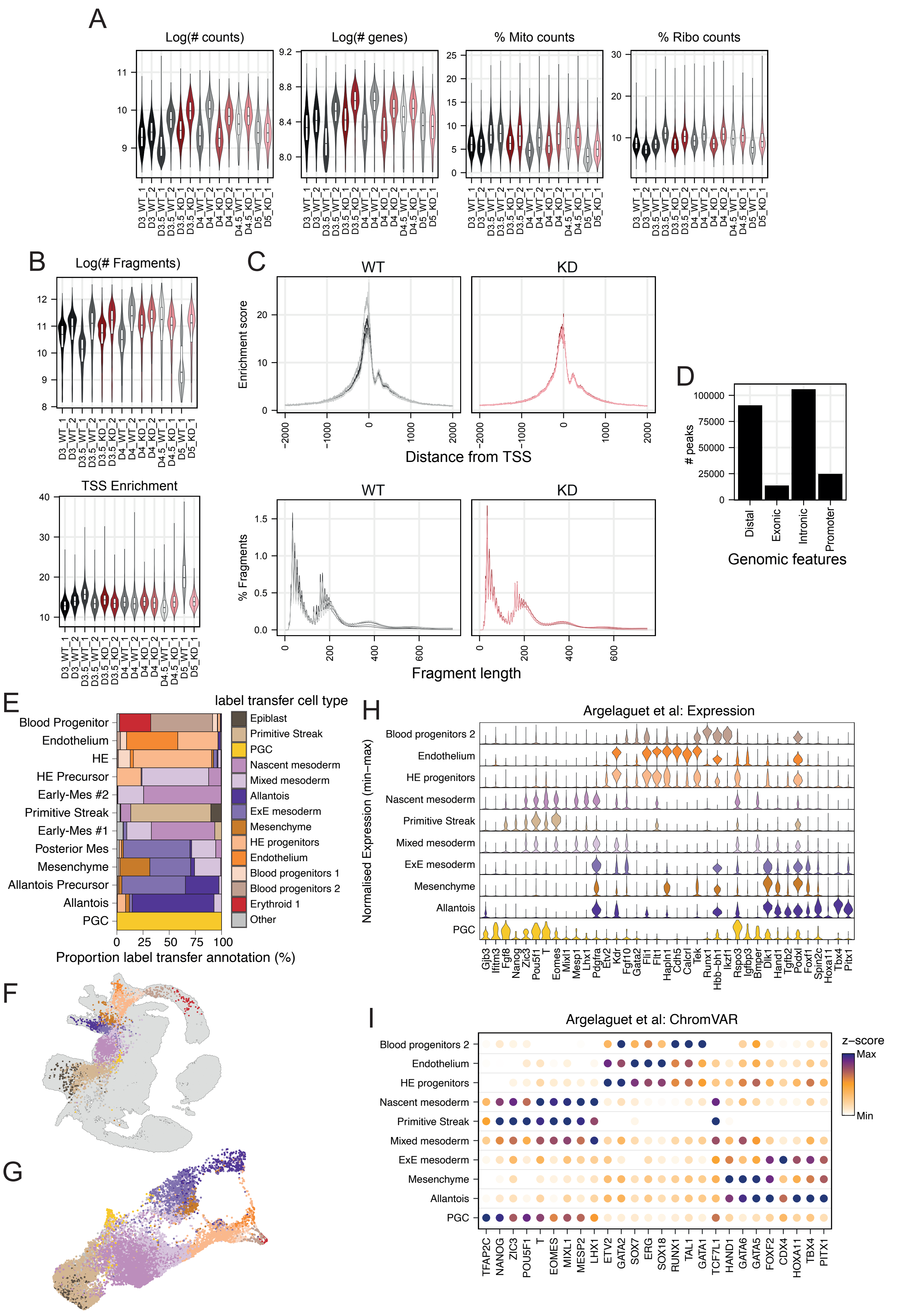

Supplementary Figure 1: Multiome quality control, related to Figure 1.

A) Violin plots displaying the natural log(number of counts), natural log(number of genes), percentage of mitochondrial and ribosomal counts per sample. WT and Eomes-KD samples are colored in shades of black and red, respectively.

B) Violin plots displaying natural log(number of fragments) and Transcription Start Sites (TSS) enrichment score, samples colored as in A.

C) Genome wide normalized enrichment score around TSS (top) and Fragment length distribution per sample (bottom), samples colored as in A.

D) Bar graph displaying number of chromatin peaks per genomic feature.

E) Transferred cell type label from integration with scRNA-seq gastrulation atlas[17] for each of the final cell type labels.

F) Projection of in vitro data on the scRNA-seq gastrulation atlas UMAP[17], colored by transferred cell type label. Cell types coloured as in E.

G) *In vitro* UMAP colored by transferred cell type label. Cell types coloured as in E.

H) Expression pattern of marker genes from Fig 1C per cell type in the *in vivo* multiome gastrulation atlas[18].

I) ChromVAR pattern of marker genes from Fig 1D per cell type in the *in vivo* multiome gastrulation atlas[18].

SUPP. FIGURE 2

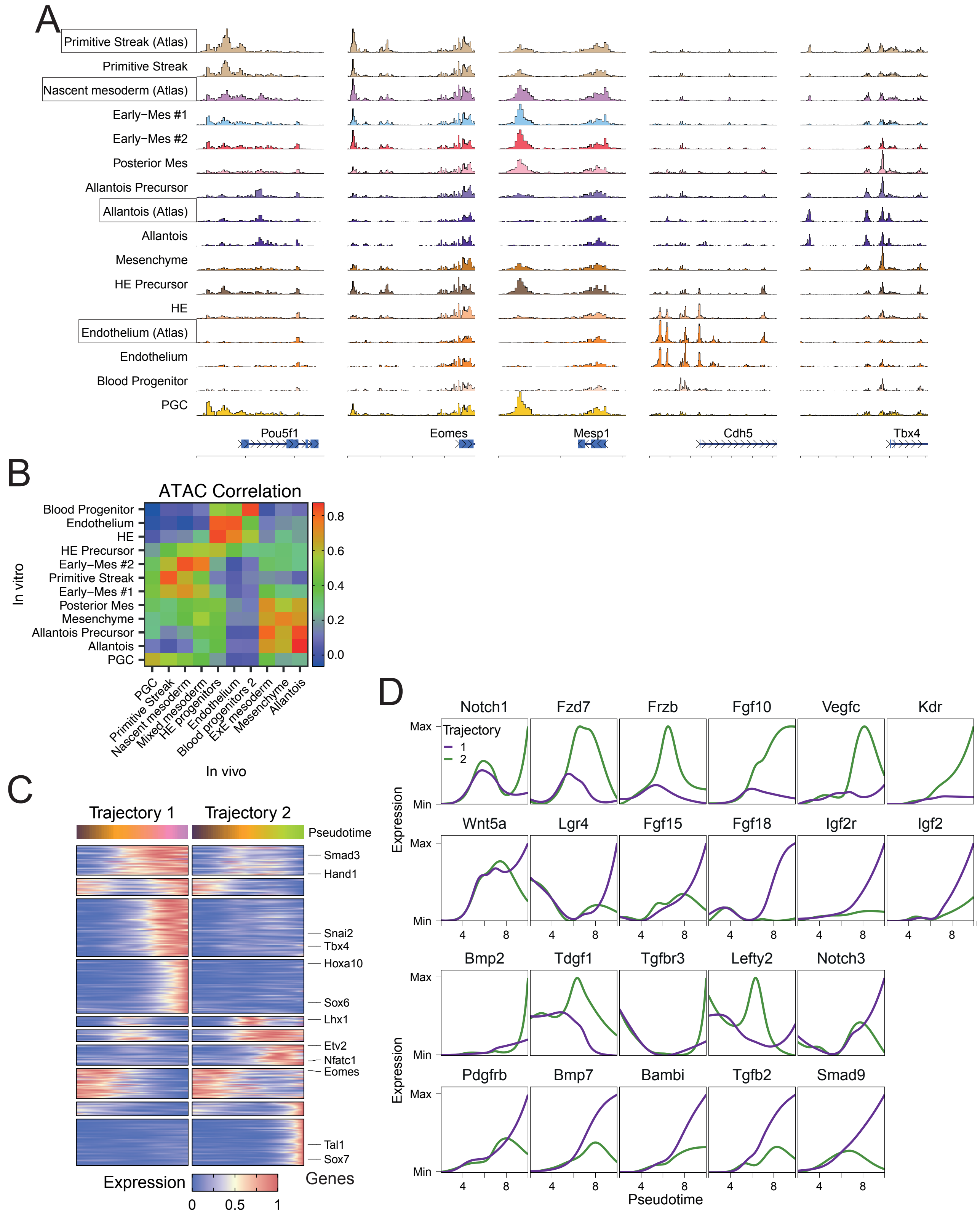

Supplementary Figure 2: EB differentiation additional information, related to Figure 1.

A) Chromatin accessibility around marker genes for PS (Pou5f1), Early Mesoderm (Eomes/Mesp1), Endothelium (Cdh5), and Allantois (Tbx4). EB cell types are compared to a subset of cell types from a mouse gastrulation *in vivo* multiomic dataset (labelled as Atlas)[18].

B) Comparison between accessibility profiles of *in vitro* cell types and *in vivo* cell types from an *in vivo* multiome gastrulation atlas[18] using cell type marker peaks identified for the *in vivo* cell types, colored by pearson correlation score.

C) Heatmap showing normalized gene expression along pseudo-time for both trajectories, clustered by expression patterns. Differential testing was performed on pseudo-time range indicated in Fig 1F.

D) Gene expression (solid lines) for allantois (purple – trajectory 1) and YS (green – trajectory 2) trajectories plotted along pseudo-time.

SUPP. FIGURE 3

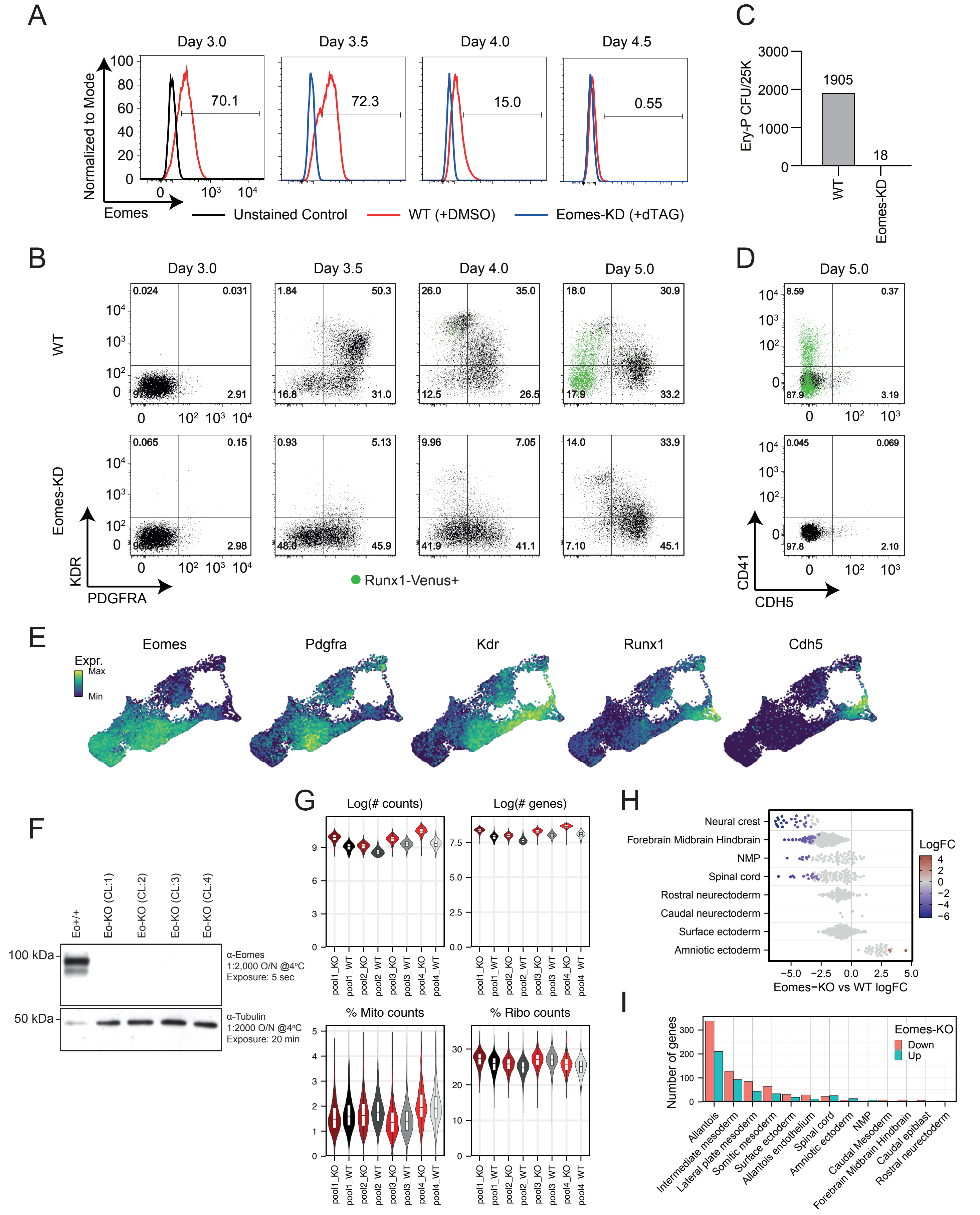

Supplementary Figure 3: Eomes-KD EB differentiation additional information, related to Figure 2 and 3.

A) Intracellular flow cytometric analyses of Eomes protein expression at different timepoints of EB differentiation. The black line at day 3 indicates the signal for the unstained control. EB cultures were treated with DMSO (red lines) and dTAG13 (blue lines) from day 3-4.

B) Flow cytometric analyses of KDR and PDGFRA expression at different timepoints of WT (top) or Eomes-KD (bottom) EB cultures. Green dots indicate Runx1-Venus+ cells.

C) Primitive erythrocyte colony forming units counted after 5 days of growth in Methocult M3434. Cells were plated at day 5 from single cell suspensions of either WT or Eomes-KD EBs. Ery-P; Primitive Erythrocytes, CFU; Colony Forming Units, 25K; 25,000 cells.

D) Flow cytometric analyses of CD41 and CDH5 expression at day 5 of WT (top) or Eomes-KD (bottom) EB culture when DMSO or dTAG13 weas added to cultures from days 3-4. Green dots indicate Runx1-Venus+ cells.

E) UMAPs of WT multiome cultures colored by expression levels of indicated genes.

F) Western blot for Eomes and α-tubulin expression in tdTomato+ Eomes (Eo) +/+ parental and four KO clones collected at day 4 of EB differentiation, confirming Eomes-KO for all four clones used in chimera-seq experiments displayed in Fig 3.

G) Violin plots displaying natural log(number of counts), natural log(number of genes), percentage of mitochondrial and ribosomal counts per sample of Eomes-KO chimera-seq, with shades of black and red indicating WT host Eomes-KO samples, respectively.

H) Differential abundance for Eomes-KO as in Fig 3D, for ectodermal lineages. Negative logFC values indicate depletion of KO cells, while positive logFC values indicate enrichment, neighborhoods without statistical significance are colored grey.

I) Number of detected differentially expressed genes that are up (blue) and down (red) regulated in Eomes-KO compared to WT for each cell type.

SUPP. FIGURE 4

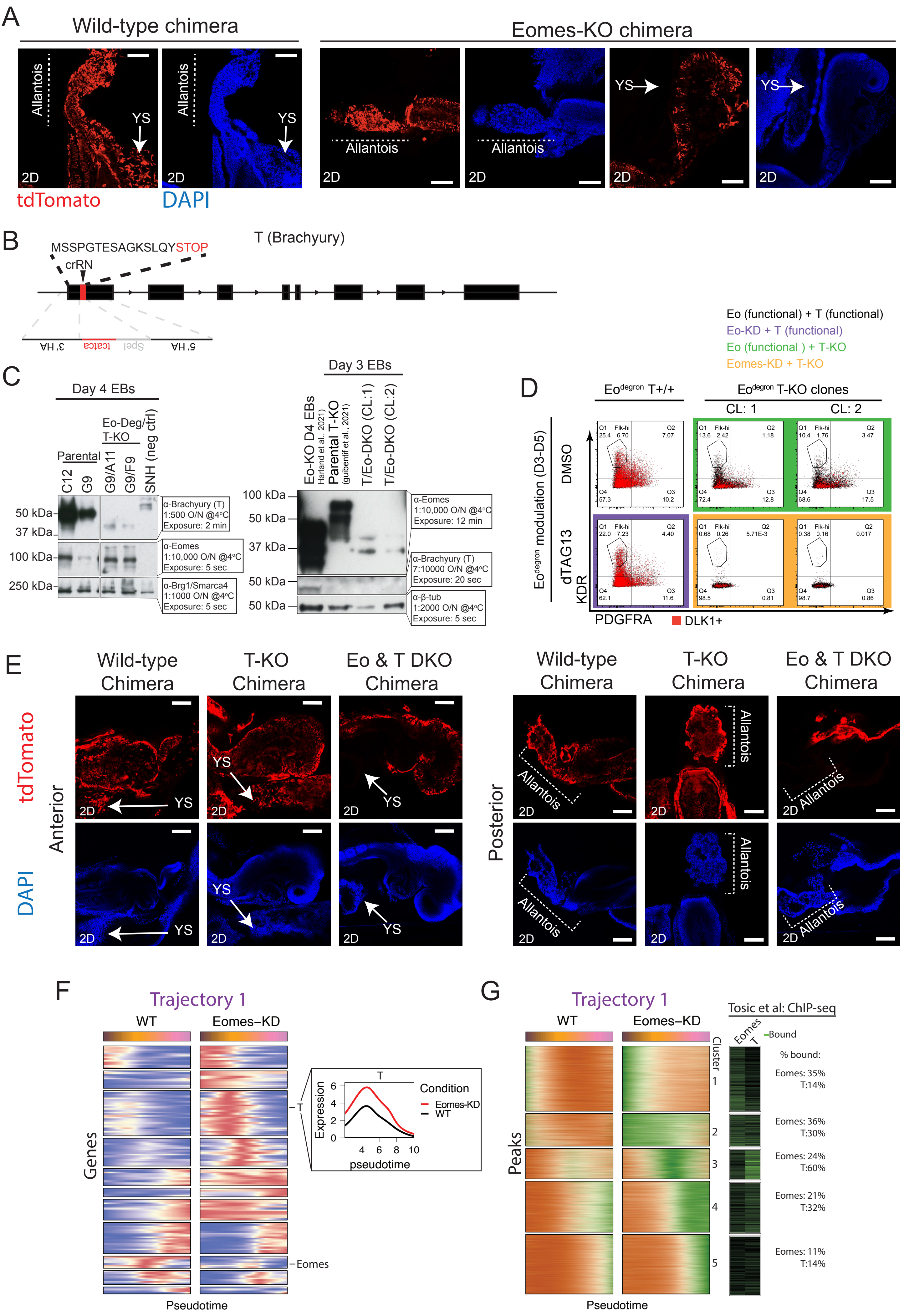

Supplementary Figure 4: Eomes and T Chimera-seq additional information, related to Figure 3 and 4.

A) Single-channel tdTomato and DAPI images of WT and Eomes Chimeric embryos from Fig 3D. Scale bars; 200 μm

B) KO strategy for T-KO used for generation of the Eo-Deg/T-KO ESC lines.

C) Western blot for T, Eomes, and Smarca4 or α-β-tubulin (loading controls) protein expression collected from day 3 and 4 EBs (as indicated) to confirm correct generation of T-KO in Eo-Deg/T-KO (left) and Eomes-KO in Eo/T-dKO lines (right).

D) Flow cytometric analyses of PDGFRA and KDR expression for $Eomes^{WT}/T^{WT}$, $Eomes^{KD}/T^{WT}$, $Eomes^{WT}/T^{KO}$, $Eomes^{KD}/T^{KO}$ at day 5 of EB differentiation. Red dots indicate DLK1 + cells. dTAG or DMSO was added from day 3 onwards.

E) Single-channel tdTomato and DAPI images from anterior (left) and posterior (right) of WT, T, and Eomes/T double KO Chimeric embryos from Fig 4D. Scale bars; 200 μm

F) Differential expression of genes between the Eomes-KD and WT conditions along the allantois trajectory (left), with normalized expression of T highlighted (right).

G) Differential accessibility of peaks between the Eomes-KD and WT conditions along the allantois trajectory (left) alongside Eomes and T ChIP seq binding to those peaks (right) from Tosic et al.[13].
